# γ-Aminobutyric Acid Alleviates Cadmium Toxicity and Is Associated with Increased Ascorbate and Glutathione Levels in *Brassica* Species

**DOI:** 10.3390/ijms27188394

**Published:** 2026-09-20

**Authors:** Xu Zeng, Meng-Qi Xu, Xin-Yue Yang, Lin-Bei Xie, Zhong-Wei Zhang, Shu Yuan

**Affiliations:** International Science and Technology Cooperation Base for Efficient Utilization of Nutrient Resources and Fertilizer Innovation, College of Resources, Sichuan Agricultural University, Chengdu 611130, China; 15974896052@163.com (X.Z.); 18708103051@163.com (M.-Q.X.); yang16970319@163.com (X.-Y.Y.); kkskado@163.com (L.-B.X.); zzwzhang@126.com (Z.-W.Z.)

**Keywords:** cadmium stress, γ-Aminobutyric acid (GABA), ascorbate–glutathione (ASA–GSH)-related metabolism, transcriptome, *Brassica* species

## Abstract

γ-Aminobutyric acid (GABA) is a signaling metabolite involved in plant stress responses; its role in cadmium (Cd) stress adaptation in oilseed crops remains poorly understood. Here, we investigated the effects of exogenous GABA on Cd stress responses in two *Brassica* species, oilseed rape (*Brassica napus*, *Bn*) and Indian mustard (*Brassica juncea*, *Bj*). GABA alleviated Cd toxicity, as indicated by improved chlorophyll retention and reduced Cd accumulation by 22–29% across the two species. Moreover, GABA altered Cd subcellular partitioning and was accompanied by elevations in ascorbate (ASA) and glutathione (GSH) contents, respectively, together with reduced superoxide anion radical (O_2_^−^) and hydrogen peroxide (H_2_O_2_) accumulation. GABA also altered fatty acid compositions, suggesting changes in membrane lipid metabolism under Cd stress. Transcriptome analysis identified 2502 and 352 upregulated genes under GABA + Cd treatment (relative to the control) in *Bn* and *Bj*, respectively, of which 2245 and 219 were uniquely detected in the GABA + Cd treatment groups. Moreover, GABA supplementation was associated with transcriptional changes in genes related to redox metabolism and ABC transporter-associated processes. Collectively, these findings suggest that GABA supplementation was associated with improved Cd tolerance in *Brassica* species, accompanied by changes in Cd subcellular partitioning, non-enzymatic antioxidant metabolism, fatty acid profiles, and stress-responsive transcriptome reprogramming.

## 1. Introduction

Cd contamination of agricultural soils has intensified with expanding industrial and agricultural activities, posing persistent risks to crop productivity, ecosystem stability, and human health because of its high mobility, persistence, and bioaccumulation potential [1,2,3,4,5]. In plants, Cd exposure triggers a comprehensive oxidative stress response characterized by the accumulation of various reactive oxygen species (ROS), including superoxide anion radical (O_2_^−^), and hydrogen peroxide (H_2_O_2_) [6]. Excess ROS induces lipid peroxidation, damages cellular membranes, and impairs essential physiological processes, including photosynthesis and nutrient metabolism [7]. To counteract oxidative damage, plants activate antioxidant defense systems, among which the ascorbate–glutathione (ASA–GSH)-related metabolism plays a central role in ROS scavenging and redox regulation. In *Brassica* species, Cd stress also enhances sulfur assimilation and glutathione metabolism by regulating genes involved in GSH biosynthesis and related detoxification processes [8,9,10].

GABA, a four-carbon non-protein amino acid, functions as both a metabolic intermediate and a signaling molecule in plants [11,12,13,14]. Under adverse conditions such as heavy metal toxicity, salinity, and drought, plants typically exhibit rapid accumulation of GABA, which contributes to enhanced stress tolerance through mechanisms including signal transduction, enhancement of antioxidant defenses, protection of the photosynthetic apparatus, and maintenance of carbon–nitrogen homeostasis [15,16,17]. Increasing evidence further indicates that exogenous GABA can alleviate Cd toxicity in several plant species by restricting Cd uptake and translocation and regulating stress-responsive metabolic pathways, including nitrogen and lipid metabolism [18,19,20]. However, whether GABA coordinates Cd compartmentalization, antioxidant metabolism, lipid homeostasis, and transcriptional responses in *Brassica* species remains poorly understood, particularly whether these responses differ between species with contrasting Cd tolerance.

Both *Bn* and *Bj* possess traits favorable for phytoremediation, including relatively high biomass production and the capacity to accumulate Cd [21,22]. However, the two species differ in their responses to Cd: *Bj* exhibits relatively higher Cd tolerance and accumulation capacity, whereas *Bn* is characterized by greater biomass production and broader adaptability to cultivation conditions. Leveraging these contrasting phenotypes, the primary objective is to uncover the mechanisms of GABA-mediated Cd tolerance in *Brassica* species. The parallel analysis of *Bn* and *Bj* serves to distinguish between conserved and species-dependent responses while identifying robust molecular processes tied to Cd adaptation. In this study, transcriptomic analyses were performed to identify GABA-responsive genes and pathways associated with antioxidant metabolism, Cd transport, and stress adaptation. Based on these findings, we further examined the effects of exogenous GABA on plant growth, chlorophyll content, Cd accumulation and subcellular distribution, ROS production, ASA–GSH metabolism, and fatty acid composition in *Bn* and *Bj* under Cd stress. The findings provide novel insights into the regulatory functions of GABA in modulating Cd accumulation and enhancing Cd tolerance in *Brassica* species.

## 2. Results

### 2.1. Transcriptomic Responses of Two Brassica Species to GABA Under Cd Stress

**Figure 1:**
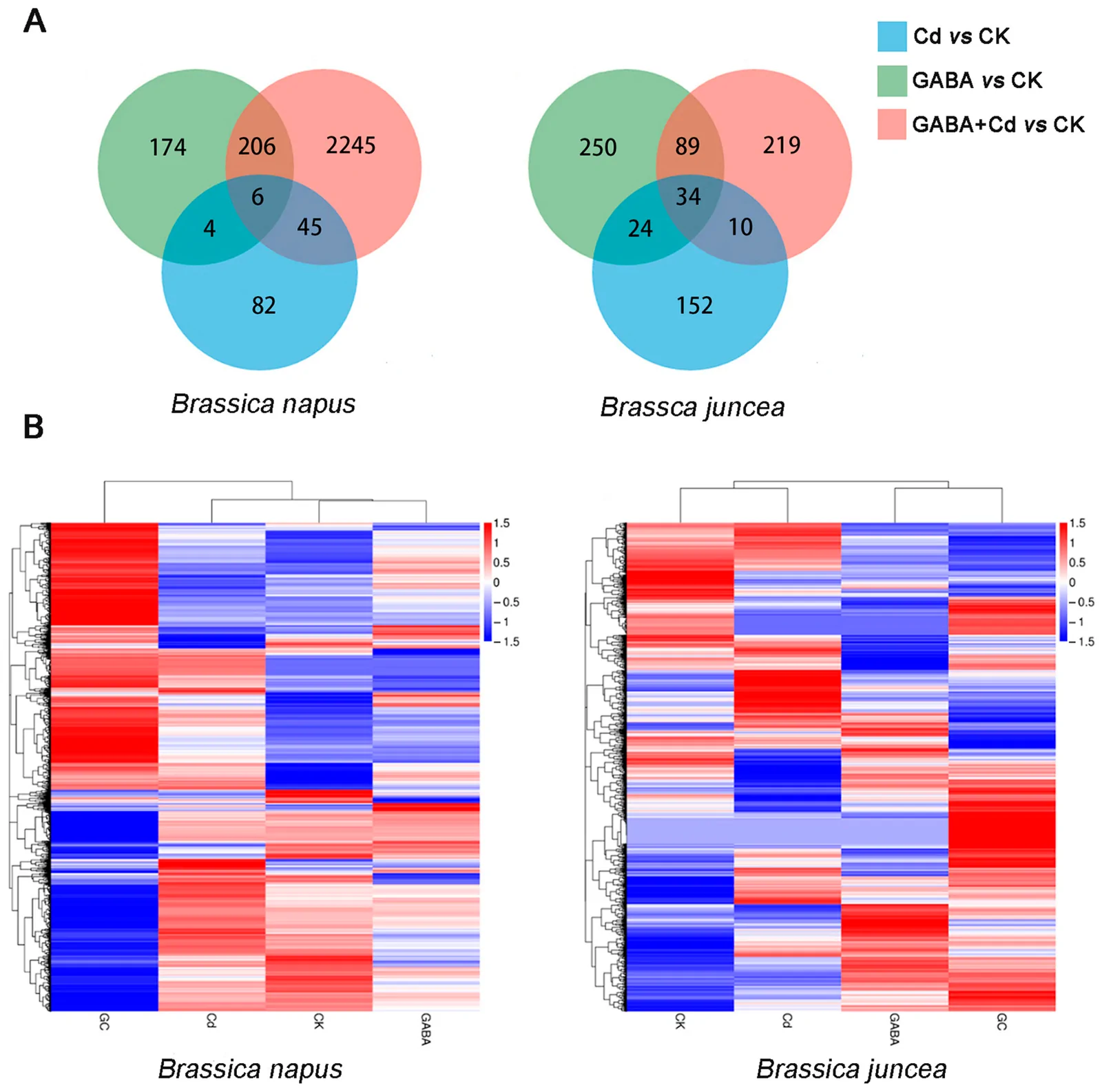
Differential gene expression in *Brassica napus (Bn)* and *Brassica juncea (Bj)* under Cd and GABA treatments. (**A**) Venn diagrams showing the distribution of upregulated differentially expressed genes (DEGs) among treatment groups. (**B**) Hierarchical clustering of DEGs in *Bn* and *Bj* under different treatments. Colors indicate relative gene expression levels from high (red) to low (blue). CK, control; GABA, 5 mM GABA; Cd, 50 μM CdCl_2_; GABA + Cd (GC), 5 mM GABA and 50 μM CdCl_2_ co-treatment.

Transcriptomic analysis revealed distinct responses to GABA and Cd treatments in the two *Brassica* species. The RNA-seq datasets generated in this study have been deposited in the NCBI Sequence Read Archive under BioProject accession number PRJNA1503244. Venn analysis of upregulated differentially expressed genes (DEGs) showed that Cd, GABA, and combined GABA + Cd treatments induced both overlapping and treatment-specific transcriptional changes in *Bn* and *Bj* (Figure 1A). In *Bn*, 82, 174, and 2245 genes were uniquely associated with the Cd vs. CK, GABA vs. CK, and GABA + Cd vs. CK comparisons, respectively. Similarly, *Bj* exhibited 152, 250, and 219 treatment-specific genes under these comparisons, of which 2245 and 219 were uniquely detected in the GABA + Cd treatment group (Supplementary Table S1). Notably, the combined GABA + Cd treatment induced a much larger number of uniquely responsive genes in *Bn* than in *Bj*. Hierarchical clustering further revealed distinct treatment-dependent expression patterns in both *Brassica* species (Figure 1B).

Gene Ontology (GO) enrichment analysis showed that GABA + Cd-responsive DEGs were mainly associated with metabolic processes, cellular processes, and stress responses in both *Brassica* species (Supplementary Figure S1). Kyoto Encyclopedia of Genes and Genomes (KEGG) analysis revealed species-specific transcriptional patterns (Supplementary Figure S2). In *Bn*, DEGs were mainly enriched in the ribosome, photosynthesis-antenna proteins, carbon metabolism, glycerolipid metabolism, and fatty acid degradation, suggesting that GABA primarily induced metabolic reprogramming and membrane-related adjustments. In contrast, *Bj* showed enrichment of glutathione metabolism, ABC transporter-related processes, starch and sucrose metabolism, glycerolipid metabolism, and fatty acid elongation, indicating stress adaptation. These results suggest that *Bn* and *Bj* employ distinct transcriptional strategies in response to GABA under Cd stress.

### 2.2. Verification of Gene Expression by qRT-PCR

**Figure 2:**
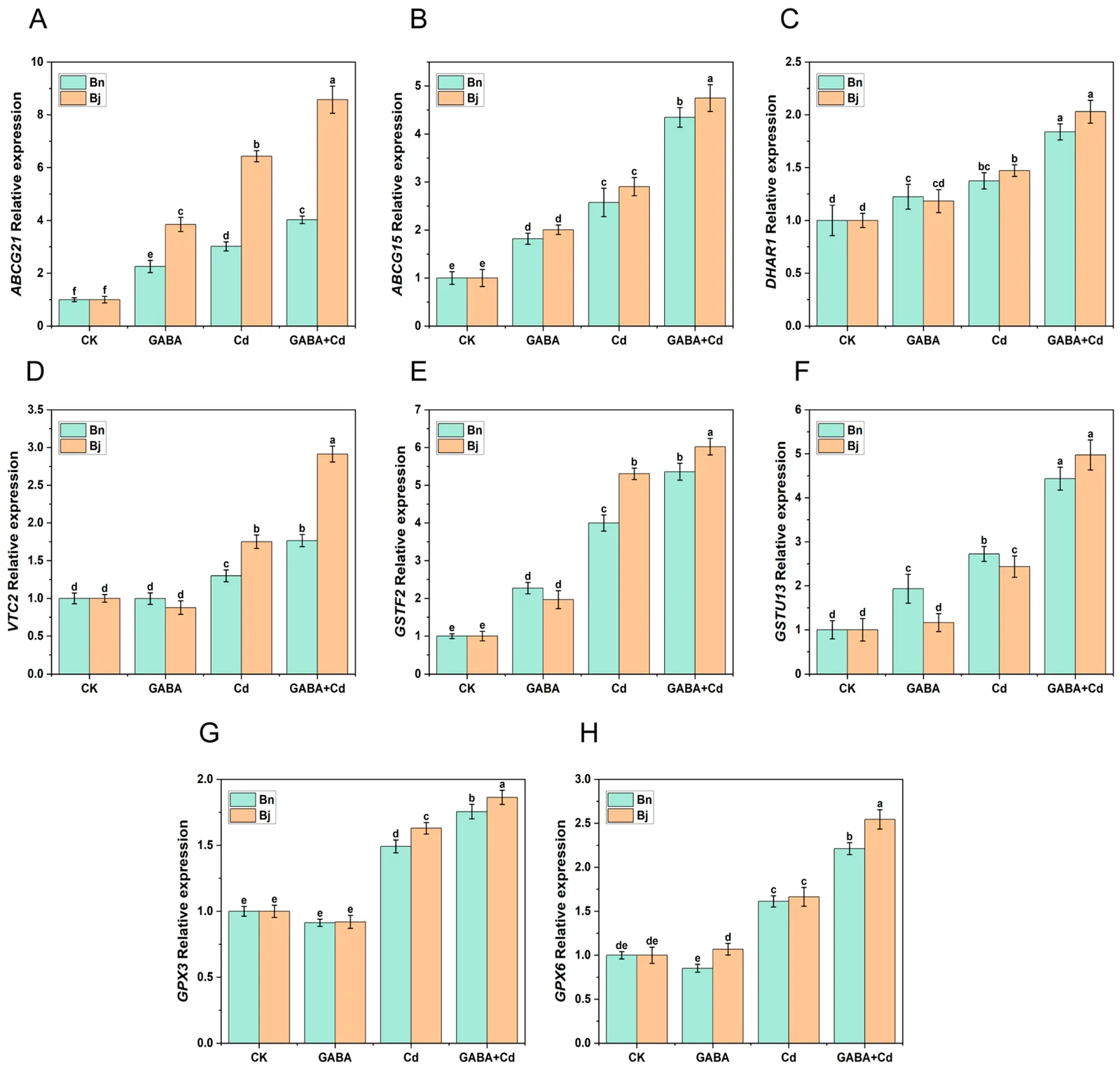
Effects of GABA on the expression of genes associated with ABC transporters (**A**,**B**), ascorbate metabolism (**C**,**D**), and glutathione metabolism (**E**–**H**) in two *Brassica* species under Cd stress. *Bn*, *Brassica napus*; *Bj*, *Brassica juncea*; CK, control; GABA, 5 mM GABA; Cd, 50 μM CdCl_2_; GABA + Cd, 5 mM GABA and 50 μM CdCl_2_ co-treatment. gene expression levels were normalized to those of the CK group, which was assigned a value of 1. Error bars show standard deviations (*n* = 3). Different lowercase letters indicate significant differences among all treatment × species combinations according to one-way ANOVA followed by Duncan’s multiple range test (*p* ≤ 0.05).

Eight representative DEGs associated with antioxidant defense, redox regulation, and Cd detoxification were selected for qRT-PCR validation. Relative expression levels were normalized to those of the CK group. The qRT-PCR expression patterns were generally consistent with the transcriptomic data (Figure 2). Under Cd stress, GABA supplementation further enhanced the Cd-induced expression of these genes. Among the tested genes, *ABCG21* and *ABCG15* exhibited the most pronounced transcriptional responses. *DHAR1* (dehydroascorbate reductase) and *VTC2* (GDP-L-galactose phosphorylase), involved in ascorbate recycling and biosynthesis, respectively, were induced by Cd and further upregulated by GABA supplementation. Similar induction patterns were observed for glutathione S-transferase genes (*GSTF2* and *GSTU13*), which participate in glutathione-dependent detoxification, and glutathione peroxidase genes (*GPX3* and *GPX6*), which are involved in ROS scavenging. Overall, *Bj* showed greater relative induction of several target genes than *Bn* under GABA + Cd treatment.

### 2.3. Effects of GABA on ASA–GSH Metabolism in Two Brassica Species Under Cd Stress

**Figure 3:**
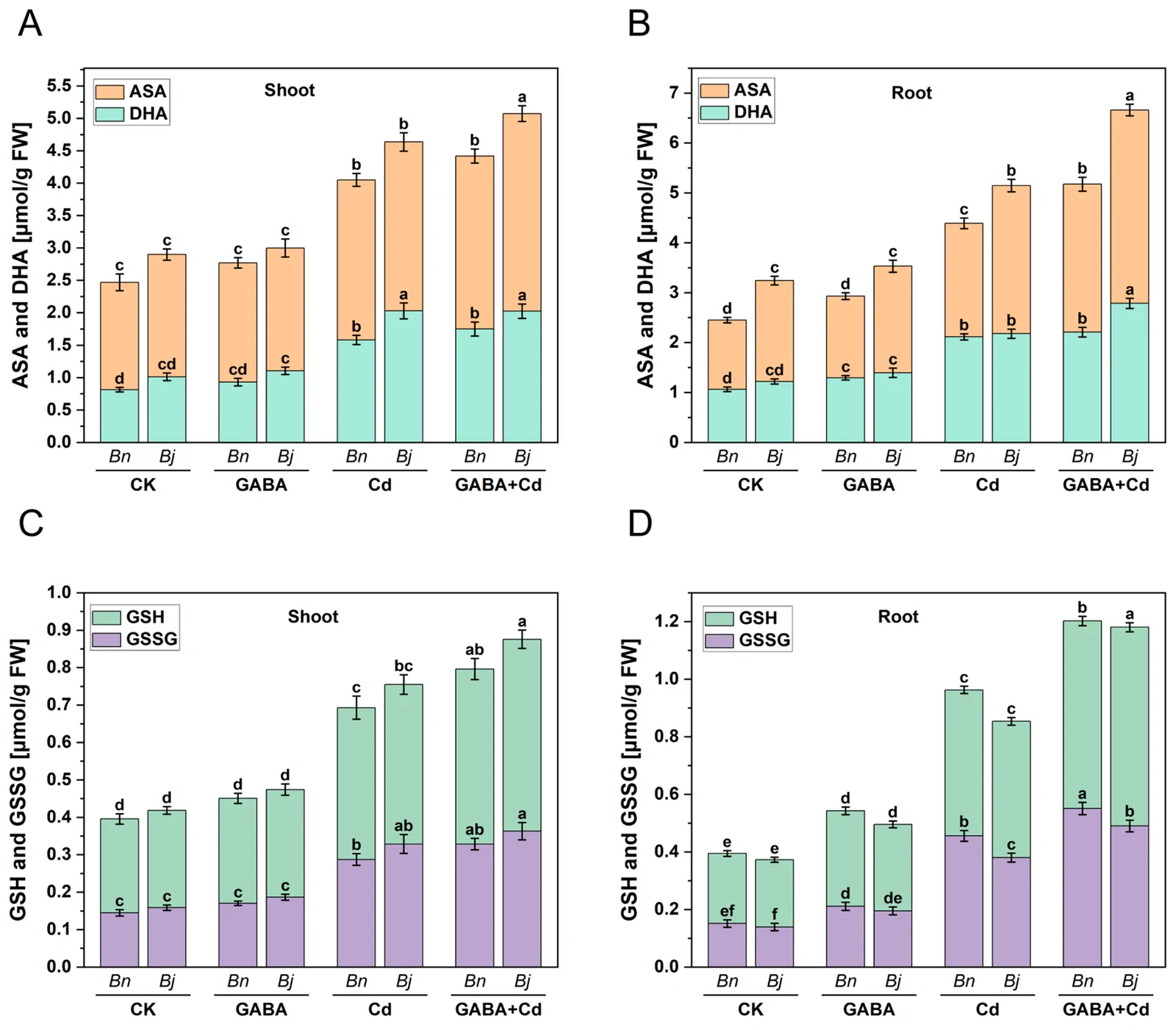
Effects of GABA on ASA–GSH antioxidant system in two *Brassica* species under Cd stress. ASA and DHA contents in shoots (**A**) and roots (**B**). GSH and GSSG contents in shoots (**C**) and roots (**D**). ASA, ascorbate; DHA, dehydroascorbate; GSH, glutathione;GSSG, glutathione disulfide, *Bn*, *Brassica napus*; *Bj*, *Brassica juncea*; CK, control; GABA, 5 mM GABA; Cd, 50 μM CdCl_2_; GABA + Cd, 5 mM GABA and 50 μM CdCl_2_ co-treatment. Bars represent means ± SD (*n* = 3). Different lowercase letters indicate significant differences among all treatment × species combinations according to one-way ANOVA followed by Duncan’s multiple range test (*p* ≤ 0.05).

Cd stress significantly altered the ASA–GSH-related redox metabolism in both *Brassica* species, resulting in increased levels of ASA, DHA, GSH, and GSSG in both shoots and roots (Figure 3). In *Bn,* compared with Cd treatment alone, exogenous GABAincreased ASA and GSH contents by 8.03–30.14% and 15.37–28.39%, while DHA and GSSG levels increased by 4.56–20.86%. In *Bj*, exogenous GABA increased ASA and GSH contents by 16.94–30.54% and 20.25–45.85%, accompanied by 0.25–28.95% increases in DHA and GSSG, respectively. In addition, GABA application showed increasing trends in the total ASA and GSH pools under Cd stress, whereas the detailed changes in ASA/DHA and GSH/GSSG ratios are presented in Supplementary Figure S3.

Consistent with the changes in ASA–GSH-related metabolites, GABA significantly increased the activities of GR and DHAR, two enzymes involved in ASA–GSH-related redox regulation, in both *Brassica* species. GR activity showed different response patterns between *Bj* and *Bn*, whereas DHAR activity differed little between the two species (Supplementary Figure S4).

**Figure 4:**
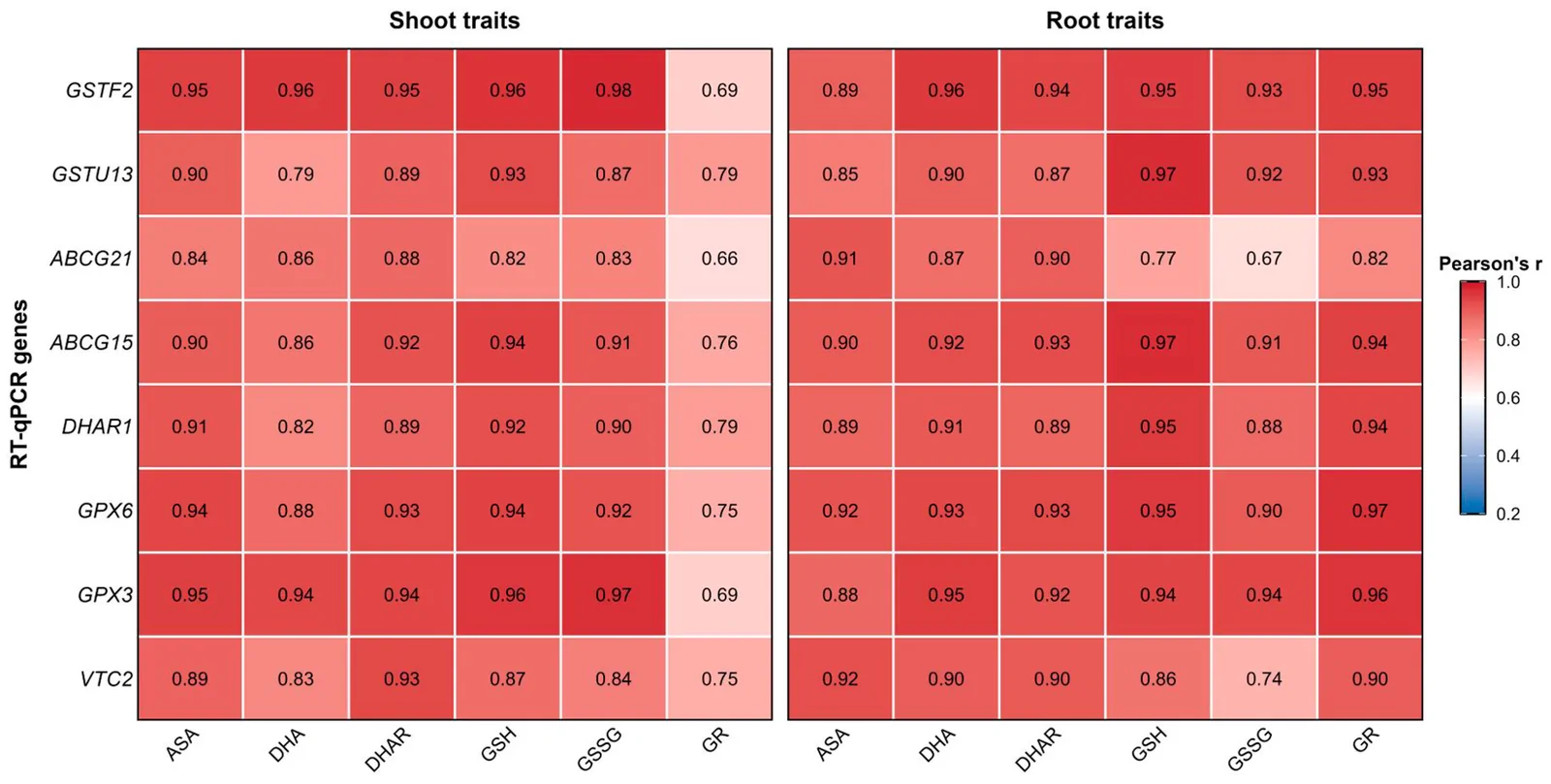
Association of candidate gene expression with ASA–GSH-related metabolism-related traits in shoots and roots. Heatmaps showing Pearson’s correlation coefficients (r) between the RT-qPCR expression levels of eight selected genes (*GSTF2*, *GSTU13*, *ABCG21*, *ABCG15*, *DHAR1*, *GPX6*, *GPX3*, and *VTC2*) and antioxidant-related traits measured in shoots and roots. Values within individual cells represent Pearson’s r. Correlation strength is represented by the color gradient, with dark red indicating stronger positive correlations and blue indicating weaker correlations. ASA, ascorbic acid; DHA, dehydroascorbate; DHAR, dehydroascorbate reductase; GSH, reduced glutathione; GSSG, oxidized glutathione; GR, glutathione reductase.

In the present study, exogenous GABA increased total ASA and GSH contents under Cd stress, indicating improved cellular redox balance. Consistent with these physiological changes, the induction of *DHAR1*, *VTC2*, *GSTF*, *GSTU* and *GPX* genes was associated with altered ASA–GSH-related metabolism observed physiologically. Meanwhile, GABA significantly increased GR and DHAR activities, suggesting that GABA may regulate ASA–GSH-related redox processes under Cd stress. RT-qPCR showed a strong correlation with the activities of key enzymes (DHAR, GR) and metabolites (ASA, DHA, GSH, GSSG) in the ASA-GSH-related metabolism (Figure 4).

### 2.4. Effects of GABA on Plant Growth, Cd Accumulation, and Cd Subcellular Distribution in Two Brassica SpeciesUnder Cd Stress

**Figure 5:**
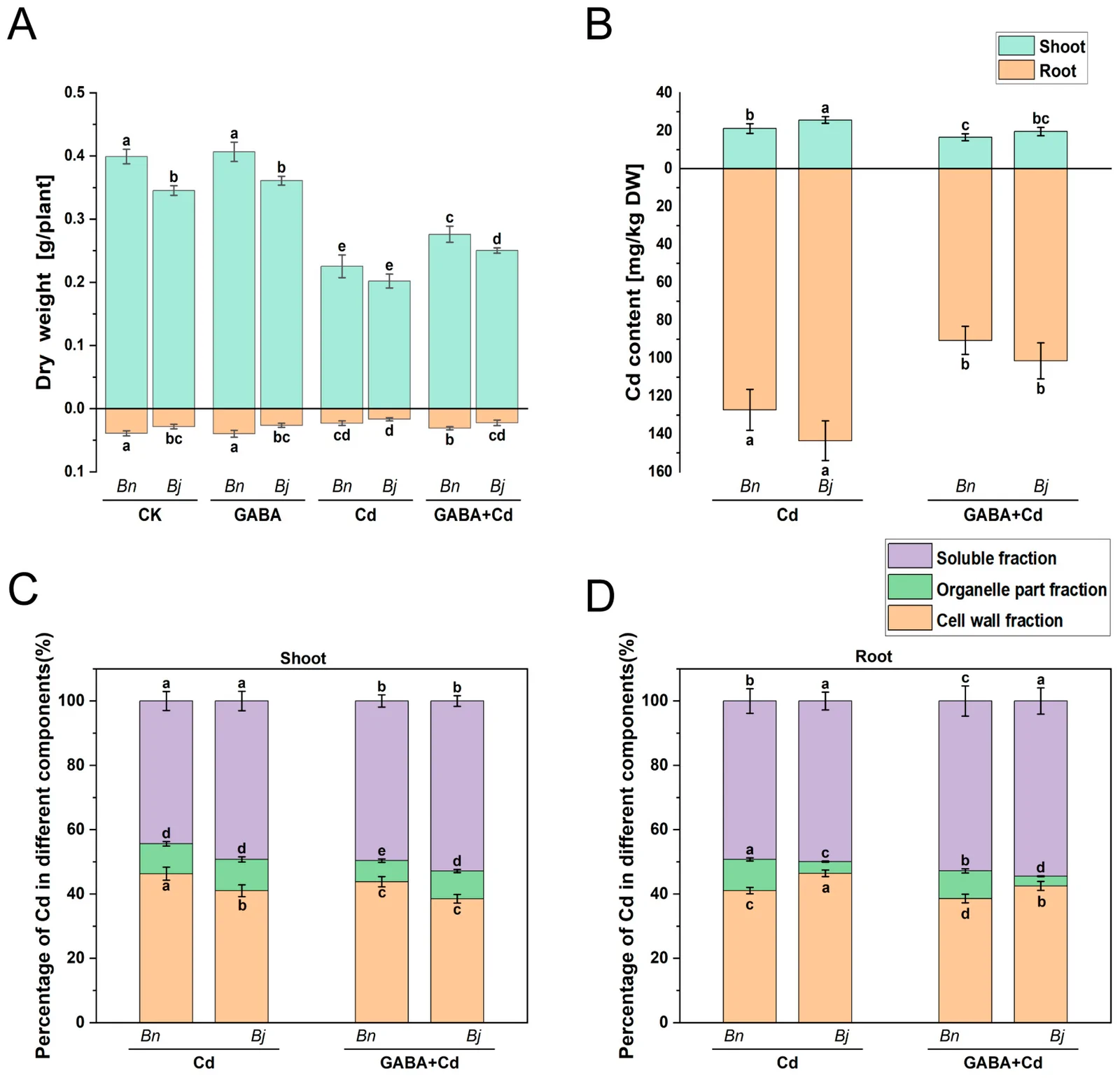
Effects of exogenous GABA ondry weight, Cd content and the subcellular distribution of Cd in two *Brassica* species under Cd stress. Shoot and root dry weights (**A**), Cd contents (**B**), and subcellular Cd distribution in shoots (**C**) and roots (**D**). CK, control; GABA, 5 mM GABA; Cd, 50 µM CdCl_2_; GABA + Cd, 5 mM GABA and 50 µM CdCl_2_ co-treatment. DW: dry weight. Bars represent means ± SD (*n* = 3). Different lowercase letters indicate significant differences among all treatment × species combinations according to one-way ANOVA followed by Duncan’s multiple range test (*p* ≤ 0.05).

**Figure 6:**
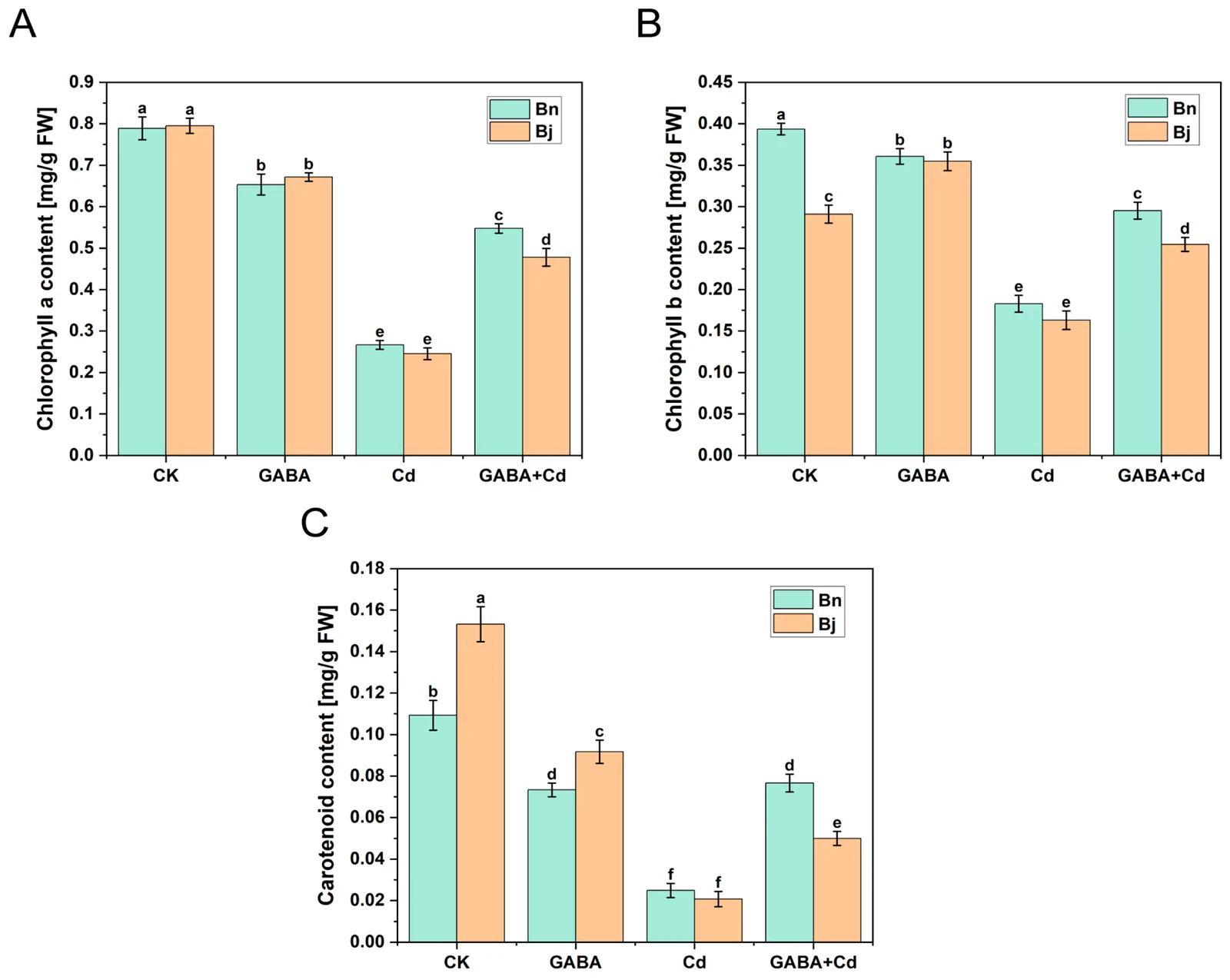
The effect of exogenous GABA on the content of photosynthetic pigments in two *Brassica* species under Cd stress. (**A**) Chlorophyll a content. (**B**) Chlorophyll b content. (**C**) Carotenoid content. *Bn*, *Brassica napus*; *Bj*, *Brassica juncea*; CK, control; GABA, 5 mM GABA; Cd, 50 μM CdCl_2_; GABA + Cd, 5 mM GABA and 50 μM CdCl_2_ co-treatment. Bars represent means ± SD (*n* = 3). Different lowercase letters indicate significant differences among all treatment × species combinations according to one-way ANOVA followed by Duncan’s multiple range test (*p* ≤ 0.05).

Cd exposure significantly decreased biomass and photosynthetic pigment contents in both *Bn* and *Bj* (Figure 5A and Figure 6). Exogenous GABA significantly restored chlorophyll a, chlorophyll b, and carotenoid contents under Cd stress and decreased Cd accumulation by 22–27% in *Bn* and 24–29% in *Bj*, with *Bj* exhibiting higher Cd accumulation than *Bn* (Figure 5B).

To further characterize Cd distribution within plant tissues, we examined subcellular Cd partitioning in both *Brassica* species. In both shoots and roots, Cd was mainly distributed in the soluble fraction, followed by the cell wall and organelle fractions (Supplementary Table S2). GABA treatment caused only a modest redistribution of Cd. In *Bn* shoots, the soluble Cd fraction increased by 5.3%, whereas the cell wall and organelle fractions decreased by 2.5% and 2.8%, respectively. Similar changes were observed in *Bj* shoots, with a 5.8% increase in the soluble fraction and decreases of 3.7% and 2% in the cell wall and organelle fractions, respectively (Figure 5C,D). Similar but smaller changes were observed in the roots. These results suggest that GABA treatment was associated with changes in Cd subcellular distribution, although the magnitude of these shifts was relatively limited compared with the reduction in total Cd accumulation.

### 2.5. GABA Alleviates Oxidative Damage in Two Brassica Species Under Cd Stress

**Figure 7:**
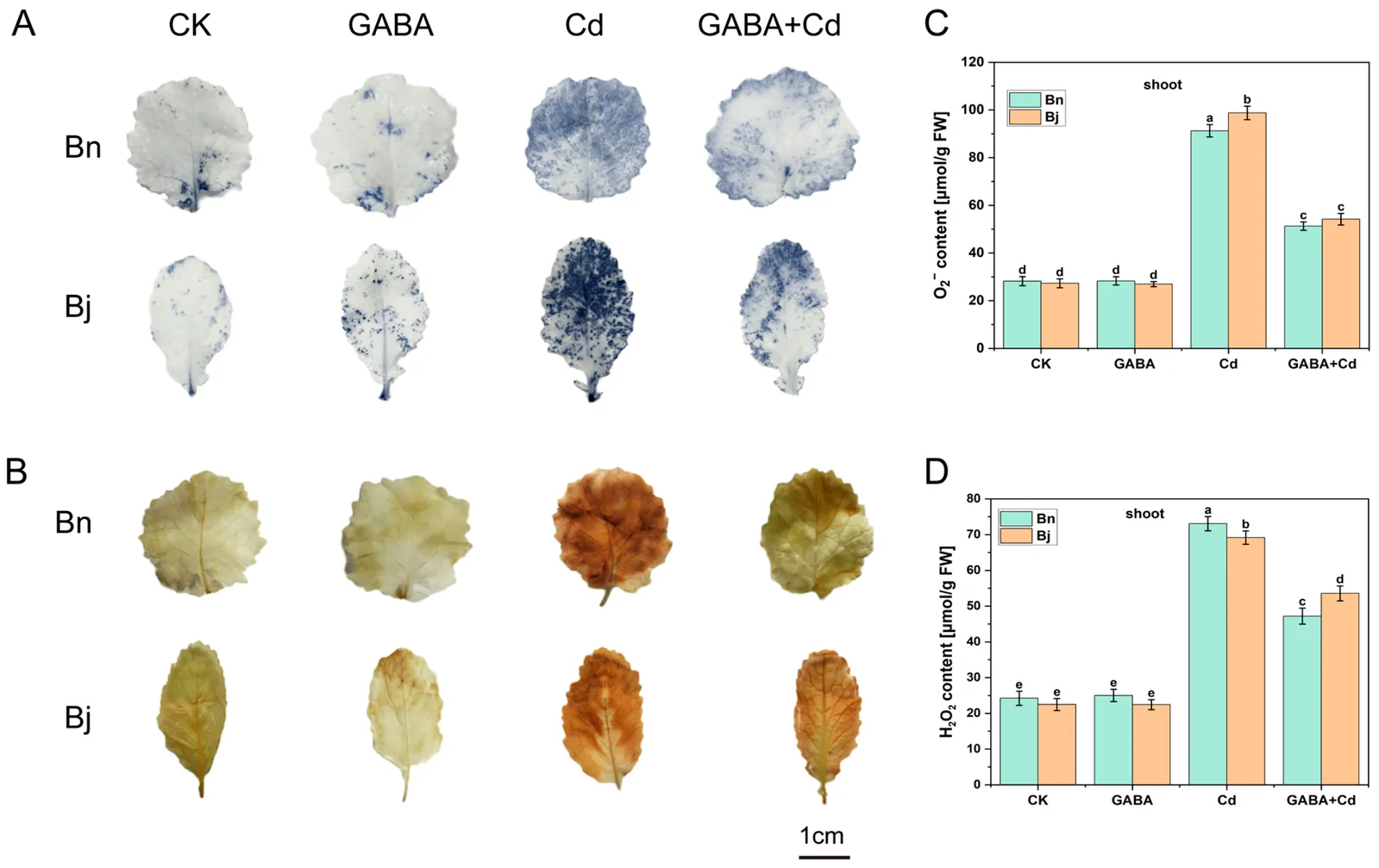
Effects of GABA on oxidative damage parameters in two *Brassica* species under Cd stress. O_2_^−^ (**A**) and H_2_O_2_ (**B**) staining in shoots, Bars = 1 cm. (**C**) O_2_^−^ content in shoots. (**D**) H_2_O_2_ content in shoots. *Bn*, *Brassica napus*; *Bj*, *Brassica juncea*; CK, control; GABA, 5 mM GABA; Cd, 50 μM CdCl_2_; GABA + Cd, 5 mM GABA and 50 μM CdCl_2_ co-treatment. FW: fresh weight. Bars represent means ± SD (*n* = 3). Different lowercase letters indicate significant differences among all treatment × species combinations according to one-way ANOVA followed by Duncan’s multiple range test (*p* ≤ 0.05).

Cd stress significantly increased H_2_O_2_ and O_2_^−^ accumulation in both *Brassica* species (Figure 7A,B). Compared with Cd treatment alone, GABA supplementation reduced O_2_^−^ levels by 45.1% and 43.8% and H_2_O_2_ levels by 26.7% and 31.8%in shoots of *Bn* and *Bj* (Figure 7C,D). Cd also induced substantial proline accumulation, whereas GABA supplementation partially alleviated this response (Supplementary Figure S5). Consistent with the ROS changes, relative electrical conductivity and malondialdehyde (MDA) content increased under Cd stress but declined following GABA supplementation (Supplementary Figure S6).

### 2.6. Exogenous GABA Alleviates Cd-Induced Changes in Fatty Acid Composition in Two Brassica Species

Cd stress altered leaf fatty acid composition in both *Brassica* species (Table 1), primarily characterized by decreased α-linolenic acid and increased linoleic and saturated fatty acids. α-Linolenic acid decreased from 54.5% to 45.5% in *Bn* and from 54.6% to 48.9% in *Bj* under Cd stress. GABA supplementation partially counteracted these changes, increasing α-linolenic acid to 49.38% and 51.60% in *Bn* and *Bj*, respectively, while the Cd treatment induced accumulation of linoleic and saturated fatty acids.

## 3. Discussion

Cd stress has been demonstrated to inhibit the growth of various crops, including rice, wheat, and *Brassica* species [23,24,25]. Cd-induced ROS accumulation and lipid peroxidation disrupt cellular redox and membrane homeostasis, representing major components of Cd toxicity in plants [26,27,28,29,30,31]. GABA is recognized as a critical signaling metabolite that modulates plant adaptation to environmental stresses, including heavy metal toxicity. Previous research has demonstrated that GABA can alleviate the toxic effects of arsenic and chromium by modulating ion transport mechanisms and enhancing antioxidant defense systems [32,33]. Similar protective effects have also been reported under low-temperature stress, hypoxia, and salinity [34,35]. Recent transgenic evidence further supports the involvement of GABA in Cd tolerance. Di et al. [36] demonstrated that *AsGAD1* overexpression increased endogenous GABA and enhanced Cd tolerance in *Arabidopsis*, accompanied by changes in Cd uptake and compartmentalization, GSH- and phytochelatin-related processes, and membrane lipid remodeling. These findings are consistent with the altered Cd partitioning, increased GSH levels, and fatty acid changes observed following exogenous GABA supplementation in the present study.

The subcellular distribution of Cd is closely associated with its toxicity in plants. After entering root cells, Cd can be retained in the cell wall or redistributed among intracellular compartments, thereby affecting the amount of Cd that reaches metabolically active sites. Notably, the cell wall and the soluble fraction frequently serve as critical sites for buffering and detoxification. In various plant species, including oilseed rape, rice, peanut, and *Arabidopsis*, Cd predominantly accumulates in root cell wall components and the soluble fraction [37,38,39,40]. This pattern indicates that immobilization of Cd within the cell wall and sequestration into vacuoles are essential strategies to limit cytosolic Cd accumulation and mitigate Cd-induced phytotoxicity.

Plants cope with Cd toxicity through coordinated regulation of mechanisms such as Cd efflux and compartmentalization [41,42]. In this study, because GABA was applied only to the foliage, the reduced Cd content in roots was unlikely to result from direct GABA–Cd interactions in the nutrient solution. The decreased whole-plant Cd content suggests that GABA affects Cd uptake or long-distance translocation, while the altered subcellular Cd distribution reflects changes in intracellular Cd sequestration and detoxification. Accordingly, reduced Cd acquisition and altered Cd partitioning may represent adaptive responses that limit Cd exposure at metabolically active sites, thereby improving physiological performance under Cd stress. Consistent with this, GABA supplementation altered the expression of two ABCG subfamily genes, *ABCG21* and *ABCG15*, under Cd stress, and the GABA-induced changes in Cd subcellular distribution suggest that its protective effect may involve not only enhanced antioxidant capacity but also Cd redistribution toward less metabolically active sites. These transcriptional changes, together with the reduced Cd accumulation at the whole-plant level, suggest that GABA may influence Cd transport processes, although the specific roles of these transporters and the underlying mechanism require further validation. Notably, the lower number of DEGs in *Bj* may reflect its higher intrinsic Cd tolerance, consistent with its stronger Cd tolerance and higher basal salicylic acid and glutamate levels [43]. This suggests that *Bj* requires fewer transcriptional adjustments to maintain homeostasis under Cd stress, possibly due to a more robust constitutive antioxidant system. Although the heterologous reference genome may have led to a conservative DEG estimate, the overall trend supports this interpretation.

Within plant cells, the levels of ROS are carefully controlled by antioxidant systems to maintain redox homeostasis. ASA and GSH represent the primary non-enzymatic antioxidants [44,45], and the ASA–GSH-related metabolism is widely recognized as a critical pathway for ROS detoxification and redox regulation during abiotic stress [46,47,48,49]. Elevated GSH concentrations have been associated with enhanced Cd tolerance in various plant species [50,51,52]. However, because other key enzymes involved in this pathway, such as ascorbate peroxidase (APX) and monodehydroascorbate reductase (MDHAR), were not examined, further functional validation is required to determine the precise contribution of ASA–GSH-related metabolism to GABA-mediated Cd tolerance.

**Figure 8:**
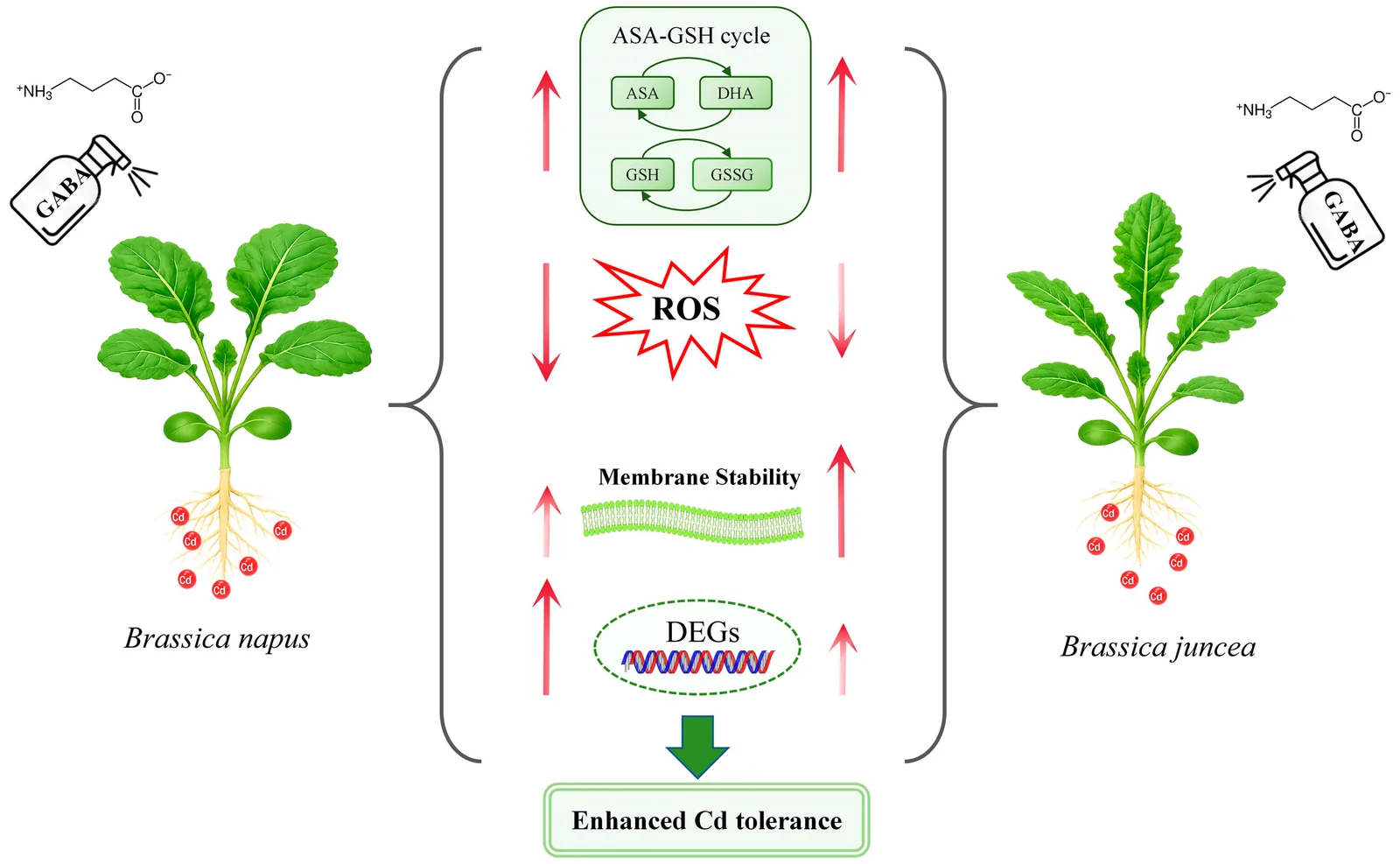
Schematic illustration of the proposed physiological and molecular mechanisms underlying enhanced cadmium (Cd) tolerance in *Brassica napus* and *Brassica juncea*. GABA treatment was associated with a reduction in reactive oxygen species (ROS) accumulation, which is consistent with changes in the activity of the ASA–GSH-related metabolism. Furthermore, the study found that GABA is involved in maintaining membrane lipid homeostasis under cadmium exposure, although the underlying mechanisms remain to be elucidated. These observations suggest that GABA-induced changes in cadmium tolerance may involve the coordinated regulation of antioxidant capacity, cadmium accumulation, and metabolic processes, with different patterns of association observed between *Bn* and *Bj*. The length of the arrows and the shade of color reflect the relative strength of the observed associations and do not imply a direct causal relationship.

Cd-induced oxidative stress can also disturb membrane lipid homeostasis in *Brassica* species by modifying fatty acid composition [53,54,55]. Notably, GABA supplementation partially reversed these Cd-induced changes in fatty acids, together with the alleviation of oxidative stress. These responses suggest that GABA supplementation may be associated with the maintenance of cellular redox status and membrane lipid homeostasis under Cd stress [56,57]. In addition, proline accumulation is generally considered an adaptive response to abiotic stress, contributing to osmotic adjustment and ROS scavenging. However, proline levels may also reflect the extent of stress-induced metabolic disturbance. In this study, the partial reduction in Cd-induced proline accumulation by GABA may indicate alleviated cellular stress rather than reduced stress tolerance. The lack of significant changes in the GABA-only treatment indicates that GABA itself did not substantially disturb cellular metabolism under normal conditions, but instead exerted its protective function mainly under Cd stress. Collectively, our findings suggest that GABA-mediated Cd tolerance in two *Brassica* species is associated with changes in antioxidant metabolism, particularly ASA–GSH metabolism, accompanied by alterations in Cd accumulation, subcellular Cd distribution, and membrane lipid profiles (Figure 8). However, the relative contribution and causal relationships among these processes remain to be determined.

## 4. Materials and Methods

### 4.1. Plant Materials, Culture Conditions

The seeds of two cultivars, ‘Zhongshuang No. 11’ (*Brassica napus*, *Bn*) and ‘Gaoxin No. 13’ (*Brassica juncea*, *Bj*), were sterilized and cold-imbibed at 4 °C for 24 h. Germination was carried out in darkness on moist filter paper at 25 °C under high-humidity conditions. After germination, uniformly sized seedlings were moved to a modified Hoagland solution and grown in a controlled environment with a photoperiod of 16 h of light and 8 h of darkness, daytime and nighttime temperatures of 22 °C and 18 °C, respectively, and 70% relative humidity. At one week post-germination, for Cd treatment, 50 µmol/L CdCl_2_ was added to the nutrient solution [31,58]. GABA (5 mmol/L) was applied by foliar spraying every 48 h at approximately 2 mL per plant until the leaves were uniformly wetted [31,58]. Control and Cd-only plants received an equal volume of distilled water. The four treatments were CK, GABA, Cd, and GABA + Cd. These four treatments were applied to both species, yielding eight treatment combinations (2 species × 4 treatments) in total. Sampling was performed 21 days after treatment initiation.

### 4.2. Determination of Cd Content

Dried samples were ground into a fine powder, and 0.1 g of each sample was accurately weighed into an acid-cleaned digestion vessel. Concentrated HNO_3_ (65%, *v*/*v*) and H_2_O_2_ (30%, *v*/*v*) were added at a volume ratio of 4:1 (5 mL in total), followed by pre-digestion at room temperature for 6 h. Samples were subsequently digested on an electric heating plate at 170 °C for 3 h until the solutions became clear. After cooling, the digests were diluted to 50 mL with ultrapure water. Total Cd concentrations were determined using an inductively coupled plasma mass spectrometer (Agilent 7900 ICP-MS, Agilent Technologies, Santa Clara, CA, USA). Cd was quantified against external calibration standards prepared from a certified Cd standard solution. Procedural blanks and quality-control samples were included during analysis to monitor contamination and analytical accuracy. Cd concentrations were expressed on a dry-weight basis (mg/kg dry weight).

### 4.3. Determination of Subcellular Cadmium Content

Differential centrifugation was employed to isolate distinct subcellular fractions from plant tissues. Specifically, 0.5 g of fresh shoot and root material was homogenized in a cold extraction buffer composed of 250 mmol/L sucrose, 50 mmol/L Tris-HCl at pH 7.5, and 1 mmol/L DL-dithioerythritol [59]. The resulting homogenate was centrifuged at 4 °C for 20 min at 4000 r/min, and the resulting pellet was classified as the crude cell wall fraction. The supernatant was then subjected to further centrifugation at 16,000 r/min for 20 min, yielding a pellet representing the organelle fraction, whilethe remaining supernatant was designated as the soluble fraction, which mainly represents soluble Cd pools including cytosolic and vacuolar components. Each fraction was subsequently evaporated to dryness and digested with HNO_3_ [60]. The digested samples were then brought to a final volume of 50 mL using deionized water, and the total cadmium concentration was determined via inductively coupled plasma mass spectrometry (Agilent 7900 ICP-MS, Santa Clara, CA, USA) [61].

### 4.4. Determination of Photosynthetic Pigment Content

The concentration of photosynthetic pigments was assessed using the method described by Lichtenthaler et al. [62]. Briefly, 0.2 g of fresh leaf tissue was ground in 10 mL of 95% ethanol inside a sealed centrifuge tube and kept in complete darkness for 24 h to allow extraction. Following decolorization, 1 mL of the supernatant was mixed with 5 mL of fresh 95% ethanol. The absorbance of the solution was then recorded at wavelengths of 665 nm, 649 nm, and 470 nm to determine the levels of chlorophyll a (Chl a), chlorophyll b (Chl b), and carotenoids, respectively.

### 4.5. Determination of Antioxidant Enzyme Activities

GSH and ASA contents were determined following established protocols [63,64]. Fresh tissue samples were immediately frozen in liquid nitrogen after harvesting and stored at −80 °C until analysis. All extraction procedures were performed rapidly on ice to minimize artificial oxidation, and samples were protected from prolonged exposure to air and light. For glutathione determination, fresh tissue samples (0.2 g) were homogenized in 5% sulfosalicylic acid and centrifuged. The supernatant was divided into two aliquots. For GSSG determination, one aliquot was treated with 1.84 mol/L triethanolamine and 10% 2-vinylpyridine to mask reduced GSH, whereas 2-vinylpyridine was replaced with distilled water for total glutathione determination. After incubation at 25 °C for 1 h, NADPH, glutathione reductase, and DTNB were added, and the formation of TNB was measured at 412 nm. Total glutathione and GSSG concentrations were calculated using standard curves generated with GSH and GSSG standards, respectively. Reduced glutathione content was calculated based on the difference between total glutathione and GSSG levels.

For ascorbate determination, 0.2 g tissue samples were homogenized in phosphate buffer (pH 7.5) under cold conditions and centrifuged at 12,000 rpm for 10 min at 4 °C. The supernatant was treated sequentially with 10 mmol/L dithiothreitol (DTT) (replaced with distilled water for reduced ASA measurement), 10% trichloroacetic acid (TCA), 44% phosphoric acid, 4% dipyridyl, and 3% ferric chloride. After incubation at 50 °C for 30 min, absorbance was measured at 520 nm. DHA content was calculated as the difference between total ASA and reduced ASA.

Glutathione reductase (GR) activity was measured following the protocol of Garcia-Limones et al. [65]. Fresh tissue was homogenized and centrifuged, and the resulting supernatant was used as the enzyme extract. GR activity was assayed in a 3.0 mL reaction mixture consisting of 100 µL enzyme extract, 100 µL of 2.4 mmol/L NADPH, 100 µL of 10 mmol/L GSSG, and 2.7 mL of 25 mmol/L potassium phosphate buffer (pH 7.0) with 2 mmol/L EDTA. The oxidation of NADPH was tracked by monitoring the decline in absorbance at 340 nm, which served as the basis for calculating enzyme activity.

Dehydroascorbate reductase (DHAR) activity was determined using the method established by Nakano and Asada [66]. DHAR activity was assayed in a 3.0 mL reaction mixture consisting of 100 µL enzyme extract, 100 µL of 2.5 mmol/L GSH, 100 µL of 0.2 mmol/L DHA, and 2.7 mL of 50 mmol/L potassium phosphate buffer (pH 7.0) containing 1 mmol/L EDTA. Ascorbate generation was monitored by recording the increase in absorbance at 265 nm. Enzyme activity was quantified from the rate of ascorbate formation, using an extinction coefficient of 14 mM^−1^ cm^−1^.

### 4.6. ROS Staining and Quantification of Oxidative Damage

The levels of superoxide (O_2_^−^) and hydrogen peroxide (H_2_O_2_) in shoots were measured using nitro blue tetrazolium (NBT) and 3,3-diaminobenzidine (DAB), respectively. Following the Cd and GABA applications, leaf tissues were immersed in either a 0.5 mg/mL NBT solution or a 1 mg/mL DAB solution for a duration of 2 h. After incubation, the plant material was cleared and destained with 80% ethanol, before being imaged with a Leica Microsystems M165 C/FC stereomicroscope (Leica Microsystems, Wetzlar, Germany) [67]. H_2_O_2_ content was determined using a Solarbio H_2_O_2_ assay kit (BC3595, Solarbio, Beijing, China) according to the manufacturer’s instructions, and expressed as μmol/g FW. O_2_^−^ content was measured using a Solarbio superoxide anion assay kit (Solarbio, Beijing, China) according to the manufacturer’s instructions, and expressed as μmol/g FW.

### 4.7. Determination of Proline, MDA, and Electrolyte Leakage

Proline content was assessed using the acid ninhydrin colorimetric approach [68]. Specifically, 0.2 g of sample was homogenized in 10 mL of 3% sulfosalicylic acid and left to extract at 4 °C for 14 h, followed by centrifugation. The resulting supernatant was then heated with ninhydrin reagent at 100 °C for 1 h and immediately chilled on ice. After partitioning with toluene, the absorbance of the organic layer was recorded at 520 nm against a toluene blank, and proline concentration was calculated from a calibration curve prepared with L-proline standards. In a separate analysis, the concentration of malondialdehyde (MDA) was measured via the thiobarbituric acid reactive substances (TBARS) assay, following the procedure of Bajji et al. [69]. Meanwhile, electrolyte leakage was evaluated according to the method of Xu et al. [70].

### 4.8. Fatty Acid Estimation

Fresh tissue samples weighing roughly 100 mg were transferred into screw-capped tubes containing 1 mL of methanol acidified with 2.5% (*v*/*v*) sulfuric acid (H_2_SO_4_). A known amount of methyl heptadecanoate (C17:0) was added as an internal standard before heating. These tubes were then heated at 80 °C for 1 h. After cooling, 1.5 mL of distilled water and 0.75 mL of hexane were introduced into the mixture. Fatty acid methyl esters (FAMEs) were partitioned into the hexane phase through vigorous shaking, and the layers were separated by centrifugation at 1500 rpm for 5 min. The hexane layer containing the methyl esters was then subjected to gas chromatographic analysis on a Hewlett-Packard 5890 Series II system (Agilent, Waldbronn, Germany). A flame ionization detector was employed, with helium used as the mobile phase [71].

### 4.9. Total RNA Extraction and Reverse Transcription, Construction of cDNA Library

Total RNA was extracted using the TRIzol (Invitrogen, Carlsbad, CA, USA) method according to the manufacturer’s instructions. RNA concentration was measured using a NanoDrop ND-2000 spectrophotometer (Thermo Fisher Scientific, Waltham, MA, USA). The eight treatment combinations (2 species × 4 treatments) described abovewere used, each with three biological replicates, resulting in 24 RNA samples. cDNA was synthesized using the K1622 RevertAid™ FAST RT Kit (Thermo Fisher Scientific) according to the manufacturer’s instructions. The cDNA libraries were sequenced by Beijing BGI Biotechnology Co., Ltd. (Beijing, China) using the Illumina sequencing platform.

### 4.10. Transcriptome Data Analysis

The sequencing platform generated image files, which were subsequently converted into raw FASTQ data using the platform’s proprietary software. All sequencing data produced in this study have been deposited in the NCBI SRA database. To obtain clean reads, adapter sequences and low-quality bases were removed from the raw datasets using fastp (v0.21.0). The resulting high-quality reads were then mapped to the *Brassica napus* reference genome (ZS.v0) with HISAT2 software [72]. For the *Brassica juncea* samples, the same *Brassica napus* reference genome (ZS.v0) was used for read mapping, as a fully annotated reference genome for *Brassica juncea* was not publicly available at the time of this analysis. Transcript abundance was quantified using StringTie (v2.1.5), which produced read count values for each gene; these counts were then used to calculate FPKM (Fragments Per Kilobase of transcript per Million mapped fragments) for normalizing gene expression levels [73]. Identification of differentially expressed genes (DEGs) was carried out using DESeq2, applying thresholds of adjusted *p*-value (*p*adj) ≤ 0.05 and an absolute log_2_ fold change ≥ 1 [74,75]. In addition, sequencing quality and base composition preferences were examined. Pairwise comparisons between sample groups were performed with DESeq2, and the resulting *p*-values were adjusted using the Benjamini–Hochberg procedure to manage the false discovery rate (FDR) [76]. DEGs were defined as those with an absolute log_2_fold change ≥ 1 and *p*adj ≤ 0.05 [77]. Functional annotation and enrichment of all DEGs were conducted using the Gene Ontology (GO) and Kyoto Encyclopedia of Genes and Genomes (KEGG) databases. GO enrichment was performed with the clusterProfiler package (version 3.8.1), where terms with *p*adj ≤ 0.05 were deemed significantly enriched [78]. Similarly, KEGG pathway enrichment analysis was executed using the clusterProfiler tool [79].

### 4.11. Quantitative Real-Time PCR Analysis

From the transcriptomic screening, a total of eight candidate genes were selected, and their transcriptional profiles were further confirmed through quantitative real-time PCR (qRT-PCR). For normalization of the qRT-PCR data, the *ACTIN* gene was used as the endogenous control. Gene-specific primers were designed with the aid of Primer Premier version 5.0 software, utilizing sequence information obtained from the NCBI database. Finally, the relative expression levels of the differentially expressed genes (DEGs) in different samples were calculated employing the comparative method (2^−ΔΔCt^) [80]. The primer sequences utilized for qRT-PCR are presented in Supplementary Table S3.

### 4.12. Statistical Analysis

For every experimental condition, a minimum of three independent biological replicates were carried out. Data analysis was conducted using SPSS (version 26.0), and comparisons among treatment groups were performed using one-way ANOVA followed by Duncan’s multiple range test. Species effects and species × treatment interactions were evaluated using two-way ANOVA, followed by Duncan’s multiple range test for post hoc comparisons of significant species, treatment, and interaction effects. Statistical significance was set at *p* ≤ 0.05.

## 5. Conclusions

In summary, transcriptomic analysis revealed that the differentially expressed genes in response to GABA + Cd in both *Brassica* species were primarily enriched in functional categories related to metabolic processes, cellular processes, and stress responses. Given the central role of glutathione metabolism in antioxidant defense, this study further conducted a systematic analysis of the ASA-GSH metabolic pathway and the activity of related antioxidant enzymes at both the physiological and molecular levels. Exogenous GABA improved Cd tolerance in *Brassica napus* and *Brassica juncea*, which was associated with changes in Cd distribution among subcellular fractions and alleviation of Cd-induced oxidative damage. GABA treatment was associated with reduced cadmium accumulation, increased ASA and GSH levels, enhanced GR and DHAR activities, and maintenance of redox homeostasis. It also partially reversed cadmium-induced changes in fatty acid composition, thereby helping to protect membrane stability. Collectively, these results provide mechanistic insights into the protective role of GABA against Cd toxicity and may provide a basis for developing GABA-based strategies to improve crop tolerance to heavy metal stress.

## Figures and Tables

**Table 1:** Effects of exogenous GABA on fatty acid composition (% of total fatty acids) in two *Brassica* species under Cd stress.

Varieties	Treatment	Oleic Acid	Linoleic Acid	α-Linolenic Acid	Palmitic Acid	Stearic Acid	Hexadecatrienoic Acid
*Brassica napus*	CK	3.4 ± 0.16 ab	15.3 ± 0.84 de	54.45 ± 1.39 a	12.63 ± 1.34 d	1.94 ± 0.22 b	12.6 ± 2.06 a
	GABA	3.37 ± 0.16 ab	16.14 ± 1.4 cd	55.48 ± 2.17 a	11.31 ± 2.11 d	2.22 ± 0.32 b	11.96 ± 1.65 a
	Cd	3.73 ± 0.19 a	21.23 ± 1.15 a	45.54 ± 1.42 c	18.45 ± 0.9 a	2.96 ± 0.19 a	8.08 ± 1.5 b
	GABA + Cd	3.33 ± 0.21 b	15.77 ± 0.81 cde	49.38 ± 2.23 bc	17.6 ± 0.65 ab	2.52 ± 0.44 ab	11.61 ± 1.09 ab
*Brassica juncea*	CK	2.46 ± 0.2 cd	14.49 ± 0.65 de	54.64 ± 1.44 a	15.46 ± 1.04 bc	1.96 ± 0.16 b	11.13 ± 2.12 ab
	GABA	2.48 ± 0.3 cd	13.67 ± 1.45 e	55.32 ± 1.99 a	14.97 ± 0.39 c	1.98 ± 0.51 b	12.06 ± 1.42 a
	Cd	2.83 ± 0.12 c	19.37 ± 1.08 ab	48.94 ± 2.17 bc	17.48 ± 1.03 ab	2.24 ± 0.12 b	9.3 ± 2.64 ab
	GABA + Cd	2.35 ± 0.18 d	17.67 ± 1.09 bc	51.6 ± 1.64 ab	15.51 ± 0.84 bc	2.2 ± 0.25 b	10.7 ± 1.13 ab

Values represent the relative abundance of individual fatty acids expressed as percentages (%) of total fatty acids. Data are presented as means ± SD (*n* = 3). Different lowercase letters indicate significant differences among all treatment × species combinations according to one-way ANOVA followed by Duncan’s multiple range test (*p* ≤ 0.05).

## Data Availability

The RNA-seq datasets generated in this study have been deposited in the NCBI Sequence Read Archive under BioProject accession number PRJNA1503244. All other data generated or analyzed during this study are included in this published article or Supplementary Materials.

## Supplementary Materials

The following supporting information can be downloaded at: https://www.mdpi.com/article/10.3390/ijms27188394/s1.

## References

[1] Lin L., Wu X., Deng X., Lin Z., Liu C., Zhang J., He T., Yi Y., Liu H., Wang Y. (2024). Mechanisms of low Cd accumulation in crops: A comprehensive overview from rhizosphere soil to edible parts. Environ. Res..

[2] Jannetto P.J., Cowl C.T. (2023). Elementary overview of heavy metals. Clin. Chem..

[3] Tang S., Yang K., Liu F., Peng M., Li K., Yang Z., Liu X., Guo F., Ma H. (2022). Overview of heavy metal pollution and health risk assessment of urban soils in Yangtze River Economic Belt, China. Environ. Geochem. Health.

[4] Rahman Z., Singh V.P. (2019). The relative impact of toxic heavy metals (THMs) (arsenic (As), Cd, chromium (Cr)(VI), mercury (Hg), and lead (Pb)) on the total environment: An overview. Environ. Monit. Assess..

[5] Chețan F., Moraru P.I., Rusu T., Șimon A., Dinca L., Murariu G. (2025). From contamination to mitigation: Addressing Cd pollution in agricultural soils. Agriculture.

[6] Rahman A., Nahar K., Hasanuzzaman M., Fujita M. (2016). Manganese-induced cadmium stress tolerance in rice seedlings: Coordinated action of antioxidant defense, glyoxalase system and nutrient homeostasis. Comptes Rendus Biol..

[7] Asgari Lajayer B., Ghorbanpour M., Nikabadi S. (2017). Heavy metals in contaminated environment: Destiny of secondary metabolite biosynthesis, oxidative status and phytoextraction in medicinal plants. Ecotoxicol. Environ. Saf..

[8] Jung H.I., Lee B.R., Chae M.J., Lee E.J., Lee T.G., Jung G.B., Kim M.S., Lee J. (2020). Ascorbate-mediated modulation of cadmium stress responses: Reactive oxygen species and redox status in *Brassica napus*. Front. Plant Sci..

[9] Mittra P.K., Rahman M.A., Roy S.K., Kwon S.J., Yun S.H., Kun C., Zhou M., Katsube-Tanaka T., Shiraiwa T., Woo S.H. (2024). Deciphering proteomic mechanisms explaining the role of glutathione as an aid in improving plant fitness and tolerance against cadmium toxicity in *Brassica napus* L. *J. Hazard*. Mater..

[10] Zhang H., Chen J., Wang W., Li Q., Zhang W., Li B., Zhang X., Hu J., Wang Z., Liu Y. (2025). Integrated transcriptomic and metabolomic analysis reveals the molecular mechanisms of cadmium stress response in rapeseed (*Brassica napus* L.): Insights into glutathione and sulfur metabolism pathways. Ind. Crops Prod..

[11] Ramesh S.A., Tyerman S.D., Gilliham M., Xu B. (2017). γ-Aminobutyric acid (GABA) signalling in plants. Cell. Mol. Life Sci..

[12] Deewatthanawong R., Rowell P., Watkins C.B. (2010). γ-Aminobutyric acid (GABA) metabolism in CO_2_-treated tomatoes. Postharvest Biol. Technol..

[13] Li L., Dou N., Zhang H., Wu C. (2021). The versatile GABA in plants. Plant Signal. Behav..

[14] Li Z., Cheng B., Peng Y., Zhang Y. (2020). Adaptability to abiotic stress regulated by γ-aminobutyric acid in relation to alterations of endogenous polyamines and organic metabolites in creeping bentgrass. Plant Physiol. Biochem..

[15] Kalhor M.S., Aliniaeifard S., Seif M., Asayesh E.J., Bernard F., Hassani B., Li T. (2018). Enhanced salt tolerance and photosynthetic performance: Implication of γ-aminobutyric acid application in salt-exposed lettuce (*Lactuca sativa* L.) plants. Plant Physiol. Biochem..

[16] Kolupaev Y.E., Shakhov I.V., Kokorev A.I., Dyachenko A.I., Dmitriev A.P. (2024). The role of reactive oxygen species and calcium ions in implementing the stress protective effect of γ-aminobutyric acid on wheat seedlings under heat stress conditions. Cytol. Genet..

[17] Konstanti P., Ligthart K., Fryganas C., Constantinos P., Smidt H., de Vos W.M., Belzer C. (2024). Physiology of γ-aminobutyric acid production by *Akkermansia muciniphila*. Appl. Environ. Microbiol..

[18] Seifikalhor M., Aliniaeifard S., Bernard F., Seif M., Latifi M., Hassani B., Didaran F., Bosacchi M., Rezadoost H., Li T. (2020). γ-Aminobutyric acid confers cadmium tolerance in maize plants by concerted regulation of polyamine metabolism and antioxidant defense systems. Sci. Rep..

[19] Zhao Y., Song X., Zhong D.B., Yu L., Yu X. (2020). γ-Aminobutyric acid (GABA) regulates lipid production and cadmium uptake by *Monoraphidium* sp. QLY-1 under cadmium stress. Bioresour. Technol..

[20] Li Y., Li Y., Cui Y., Xie Y., Shi Y., Shang Y., Ma F., Zhang J., Li C. (2022). GABA-mediated inhibition of cadmium uptake and accumulation in apples. Environ. Pollut..

[21] Cao X., Wang X., Lu M., Hamid Y., Lin Q., Liu X., Li T., Liu G., He Z., Yang X. (2021). The Cd phytoextraction potential of hyperaccumulator *Sedum alfredii*–oilseed rape intercropping system under different soil types and comprehensive benefits evaluation under field conditions. Environ. Pollut..

[22] Yuan T., Gu J., Zhou H., Huang F., Yang W., Wang S., Zhang J., Huo Y., Liao B. (2020). Translocation and accumulation of cadmium and lead in the tissues of 39 rape cultivars grown in a polluted farmland. Environ. Sci. Pollut. Res. Int..

[23] Dong Q., Wu Y., Li B., Chen X., Peng L., Sahito Z.A., Li H., Chen Y., Tao Q., Xu Q. (2023). Multiple insights into lignin-mediated Cd detoxification in rice (*Oryza sativa*). J. Hazard. Mater..

[24] Zhang D., Liu J., Zhang Y., Wang H., Wei S., Zhang X., Zhang D., Ma H., Ding Q., Ma L. (2023). Morphophysiological, proteomic and metabolomic analyses reveal Cd tolerance mechanism in common wheat (*Triticum aestivum* L.). J. Hazard. Mater..

[25] Bhardwaj T., Kour J., Chouhan R., Devi K., Singh H., Gandhi S.G., Ohri P., Bhardwaj R., Alsahli A.A., Ahmad P. (2024). Integrated transcriptomic and physio-molecular studies unveil the melatonin and PGPR induced protection to photosynthetic attributes in *Brassica juncea* L. under Cd toxicity. J. Hazard. Mater..

[26] Foyer C.H., Noctor G. (2005). Redox homeostasis and antioxidant signaling: A metabolic interface between stress perception and physiological responses. Plant Cell.

[27] Foyer C.H., Noctor G. (2011). Ascorbate and glutathione: The heart of the redox hub. Plant Physiol..

[28] Krantev A., Yordanova R., Janda T., Szalai G., Popova L. (2008). Treatment with salicylic acid decreases the effect of Cd on photosynthesis in maize plants. J. Plant Physiol..

[29] Qureshi F.F., Ashraf M.A., Rasheed R., Ali S., Hussain I., Ahmed A., Iqbal M. (2020). Organic chelates decrease phytotoxic effects and enhance chromium uptake by regulating chromium-speciation in castor bean (*Ricinus communis* L.). Sci. Total Environ..

[30] Cuypers A., Vanbuel I., Iven V., Alen M., Hendrix S. (2023). Cd-induced oxidative stress responses and acclimation in plants require fine-tuning of redox biology at subcellular level. Free Radic. Biol. Med..

[31] Zhang Z.W., Dang T.T., Yang X.Y., Ahmed M., Wang Y., Zhang G., Wu F. (2024). Gamma-aminobutyric acid alleviates programmed cell death in two *Brassica* species under Cd stress. Int. J. Mol. Sci..

[32] Kumar N., Dubey A.K., Upadhyay A.K., Sahu N., Behera S.K., Sanyal I. (2017). GABA accretion reduces *Lsi-1* and *Lsi-2* gene expressions and modulates physiological responses in *Oryza sativa* to provide tolerance towards arsenic. Sci. Rep..

[33] Mahmud J., Hasanuzzaman M., Nahar K., Bhuyan M.H.M.B., Fujita M. (2017). Gamma-aminobutyric acid (GABA) confers chromium stress tolerance in *Brassica juncea* L. by modulating the antioxidant defense and glyoxalase systems. Ecotoxicology.

[34] Salvatierra A., Pimentel P., Almada R., Hinrichsen P., Cabello A.S., Pinto M. (2016). Exogenous GABA application transiently improves the tolerance to root hypoxia in a sensitive genotype of *Prunus* rootstock. Environ. Exp. Bot..

[35] Aljuaid B.S., Ashour H. (2022). Exogenous gamma-aminobutyric acid (GABA) application mitigates salinity stress in maize plants. Life.

[36] Di Y., Cao Y., Peng D., Liu Y., Li Z. (2025). AsGAD1 cloned from creeping bentgrass modulates cadmium tolerance of *Arabidopsis thaliana* by remodelling membrane lipids and cadmium uptake, transport and chelation. Physiol. Plant..

[37] Guan M.Y., Zhang H.H., Pan W., Jin C.W., Lin X.Y. (2018). Sulfide alleviates cadmium toxicity in Arabidopsis plants by altering the chemical form and the subcellular distribution of cadmium. Sci. Total Environ..

[38] Su Y., Liu J.L., Lu Z.W., Wang X.M., Zhang Z., Shi G.R. (2014). Effects of iron deficiency on subcellular distribution and chemical forms of cadmium in peanut roots in relation to its translocation. Environ. Exp. Bot..

[39] Cao Z.Z., Qin M.L., Lin X.Y., Zhu Z.W., Chen M.X. (2018). Sulfur supply reduces cadmium uptake and translocation in rice grains (*Oryza sativa* L.) by enhancing iron plaque formation, cadmium chelation and vacuolar sequestration. Environ. Pollut..

[40] Li L., Fan Z., Gan Q., Xiao G., Luan M., Zhu R., Zhang Z. (2025). Conservative mechanism through various rapeseed (*Brassica napus* L.) varieties respond to heavy metal (Cadmium, Lead, Arsenic) stress. Front. Plant Sci..

[41] Marques D.N., Thiengo C.C., Azevedo R.A. (2025). Phytochelatins and cadmium mitigation: Harnessing genetic avenues for plant functional manipulation. Int. J. Mol. Sci..

[42] Tian J., Wang L., Hui S., Yang D., He Y., Yuan M. (2023). Cadmium accumulation regulated by a rice heavy-metal importer is harmful for host plant and leaf bacteria. J. Adv. Res..

[43] Zhang Z.W., Deng Z.L., Tao Q., Peng H.Q., Wu F., Fu Y.F., Yang X.Y., Xu P.Z., Li Y., Wang C.Q. (2022). Salicylate and Glutamate Mediate Different Cd Accumulation and Tolerance between *Brassica napus* and *Brassica juncea*. Chemosphere.

[44] Singh S., Parihar P., Singh R., Singh V.P., Prasad S.M. (2015). Heavy metal tolerance in plants: Role of transcriptomics, proteomics, metabolomics, and ionomics. Front. Plant Sci..

[45] Djanaguiraman M., Boyle D.L., Welti R., Jagadish S.V.K., Prasad P.V.V. (2018). Decreased photosynthetic rate under high temperature in wheat is due to lipid desaturation, oxidation, acylation, and damage of organelles. BMC Plant Biol..

[46] Liu S., Liu S., Wang M., Wei T., Meng C., Wang M., Xia G. (2014). A wheat similar to rcd-one gene enhances seedling growth and abiotic stress resistance by modulating redox homeostasis and maintaining genomic integrity. Plant Cell.

[47] Fotopoulos V., Ziogas V., Tanou G., Molassiotis A., Anjum N.A., Chan M.T., Sanita di Toppi L. (2010). Involvement of ASA/DHA and GSH/GSSG ratios in gene and protein expression and in the activation of defence mechanisms under abiotic stress conditions. Ascorbate-Glutathione Pathway and Stress Tolerance in Plants.

[48] Song L., Wang J., Shafi M., Liu Y., Wang J., Cao S. (2016). Hypobaric treatment effects on chilling injury, mitochondrial dysfunction, and the ascorbate-glutathione (ASA-GSH) cycle in postharvest peach fruit. J. Agric. Food Chem..

[49] Lou L., Kang J., Pang H., Li Q., Cui J., Wang S., Shen W. (2017). Sulfur protects pakchoi (*Brassica chinensis* L.) seedlings against Cd stress by regulating ascorbate-glutathione metabolism. Int. J. Mol. Sci..

[50] Lin Y.F., Aarts M.G. (2012). The molecular mechanism of zinc and Cd stress response in plants. Cell. Mol. Life Sci..

[51] Sobrino-Plata J., Carrasco-Gil S., Abadia J., Escobar C., Alvarez-Fernandez A., Hernandez L.E. (2014). The role of glutathione in mercury tolerance resembles its function under cadmium stress in *Arabidopsis*. Metallomics.

[52] Cai Y., Cao F., Wei K., Zhang G., Wu F. (2011). Genotypic dependent effect of exogenous glutathione on Cd-induced changes in proteins, ultrastructure and antioxidant defense enzymes in rice seedlings. J. Hazard. Mater..

[53] Nouairi I., Ben Ammar W., Ben Youssef N., Ben Miled Daoud D., Ghorbal M.H., Zarrouk M. (2006). Comparative study of cadmium effects on membrane lipid composition of *Brassica juncea* and *Brassica napus* leaves. Plant Sci..

[54] Møller I.M., Jensen P.E., Hansson A. (2007). Oxidative modifications to cellular components in plants. Annu. Rev. Plant Biol..

[55] Hanzel D.K., Williams J.P. (1990). Membrane lipid unsaturation and ion permeability in plants. Plant Physiol..

[56] Bouché N., Fromm H. (2004). GABA in plants: Just a metabolite?. Trends Plant Sci..

[57] Signorelli S., Coitiño E.L., Borsani O., Monza J. (2015). Molecular mechanisms for the role of GABA in plant stress responses. Plant Physiol. Biochem..

[58] Ren J., Zhu W., Sun L., Long S., Luo M., Zhou Q. (2026). Exogenous GABA enhances phenolics and maintains antioxidant capacity of blueberries by modulating the GABA shunt. Food Chem..

[59] Li X., Ma H., Li L., Gao Y., Li Y., Xu H. (2019). Subcellular distribution, chemical forms and physiological responses involved in cadmium tolerance and detoxification in *Agrocybe aegerita*. Ecotoxicol. Environ. Saf..

[60] Weigel H.J., Jäger H.J. (1980). Subcellular distribution and chemical form of cadmium in bean plants. Plant Physiol..

[61] Vogel-Mikuš K., Arčon I., Kodre A. (2010). Cd complexation in seeds and vegetative tissues of the Cd hyperaccumulator *Thlaspi praecox* as studied by X-ray absorption spectroscopy. Plant Soil.

[62] Lichtenthaler H.K., Wellburn A.R. (1983). Determinations of total carotenoids and chlorophylls a and b of leaf extracts in different solvents. Biochem. Soc. Trans..

[63] Tietze F. (1969). Enzymic method for quantitative determination of nanogram amounts of total and oxidized glutathione: Applications to mammalian blood and other tissues. Anal. Biochem..

[64] Kampfenkel K., Vanmontagu M., Inze D. (1995). Extraction and determination of ascorbate and dehydroascorbate from plant tissue. Anal. Biochem..

[65] Garcia-Limones C., Hervas A., Navas-Cortes J.A., Jimenez-Diaz R.M., Tena M. (2002). Induction of an antioxidant enzyme system and other oxidative stress markers associated with compatible and incompatible interactions between chickpea (*Cicer arietinum* L.) and *Fusarium oxysporum* f. sp. *Ciceris*. Physiol. Mol. Plant Pathol..

[66] Nakano Y., Asada K. (1981). Hydrogen peroxide is scavenged by ascorbate-specific peroxidase in spinach chloroplasts. Plant Cell Physiol..

[67] Zhang Z.W., Dong Y.Y., Feng L.Y., Deng Z.L., Xu Q., Tao Q., Wang C.Q., Chen Y.E., Yuan M., Yuan S. (2020). Selenium enhances cadmium accumulation capability in two mustard family species-*Brassica napus* and *B. juncea*. Plants.

[68] Hodges D.M., DeLong J.M., Forney C.F., Prange R.K. (1999). Improving the thiobarbituric acid-reactive-substances assay for estimating lipid peroxidation in plant tissues containing anthocyanin and other interfering compounds. Planta.

[69] Bajji M., Kinet J.M., Lutts S. (2002). The use of the electrolyte leakage method for assessing cell membrane stability as a water stress tolerance test in durum wheat. Plant Growth Regul..

[70] Xu J., Jansma S.Y., Wolters-Arts M., de Groot P.F., Jansen M.J., Rieu I. (2022). Long-term mild heat causes post-mitotic pollen abortion through a local effect on flowers. Front. Plant Sci..

[71] Le Guedard M., Schraauwers B., Larrieu I., Bessoule J.J. (2008). Development of a biomarker for metal bioavailability: The lettuce fatty acid composition. Environ. Toxicol. Chem..

[72] Kim D., Langmead B., Salzberg S.L. (2015). HISAT: A fast spliced aligner with low memory requirements. Nat. Methods.

[73] Pertea M., Kim D., Pertea G.M., Leek J.T., Salzberg S.L. (2016). Transcript-level expression analysis of RNA-seq experiments with HISAT, StringTie and Ballgown. Nat. Protoc..

[74] Anders S., Reyes A., Huber W. (2012). Detecting differential usage of exons from RNA-seq data. Genome Res..

[75] Buchfink B., Xie C., Huson D.H. (2015). Fast and sensitive protein alignment using DIAMOND. Nat. Methods.

[76] Love M.I., Huber W., Anders S. (2014). Moderated estimation of fold change and dispersion for RNA-seq data with DESeq2. Genome Biol..

[77] Varet H., Brillet-Guéguen L., Coppée J.Y., Dillies M.A. (2016). SARTools: A DESeq2 and EdgeR-based R pipeline for comprehensive differential analysis of RNA-seq data. PLoS ONE.

[78] Young M.D., Wakefield M.J., Smyth G.K., Oshlack A. (2010). Gene ontology analysis for RNA-seq: Accounting for selection bias. Genome Biol..

[79] Yu G., Wang L.G., Han Y., He Q.Y. (2012). clusterProfiler: An R package for comparing biological themes among gene clusters. Omics.

[80] Livak K.J., Schmittgen T.D. (2001). Analysis of relative gene expression data using real-time quantitative PCR and the 2^−ΔΔC_T_^ Method. Methods.

