# Supplementary material for: γ-Aminobutyric Acid Alleviates Cadmium Toxicity and Is Associated with Increased Ascorbate and Glutathione Levels in Brassica Species
Source: Int J Mol Sci. 2026 Sep 20;27(18):8394. doi: 10.3390/ijms27188394 (PMC13607661; doi:10.3390/ijms27188394)
Supplement: Supplementary file 1 [file ijms-27-08394-s001.zip › Supplementary Information.pdf]

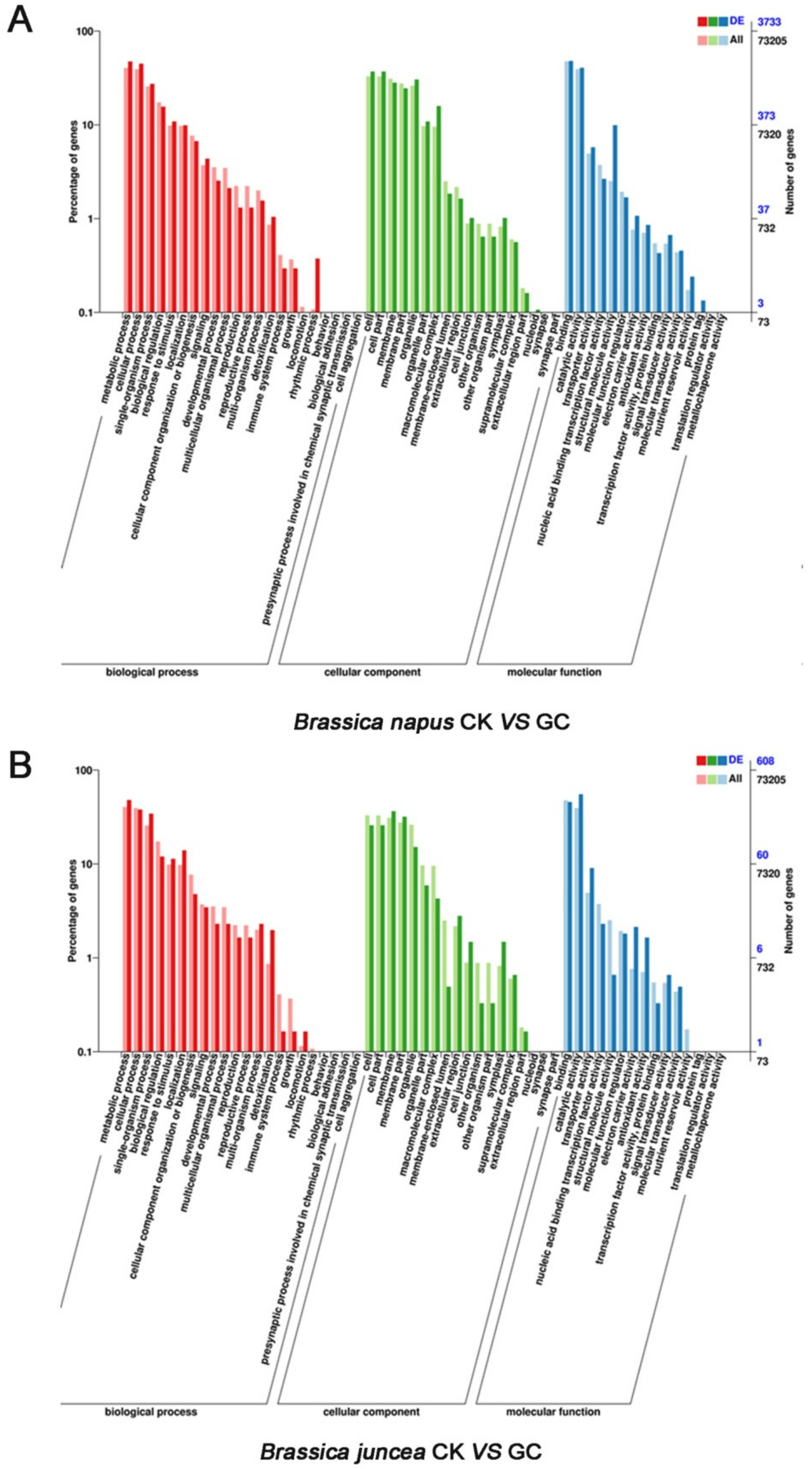

Supplementary Figure S1. GO functional classification of DEGs in *Brassica napus* and *Brassica juncea* under Cd stress and exogenous GABA application. (A) GO functional classification of DEGs under Cd stress (CK vs Cd); (B) GO functional classification of DEGs under combined GABA and Cd treatment (CK vs GC). GO terms were divided into biological process, cellular component, and molecular function. The left y-axis represents the percentage of genes assigned to specific GO terms, and the right y-axis indicates the number of genes. CK, control; GABA, 5 mM GABA; Cd, 50 μM $CdCl_2$; GABA+Cd, 5 mM GABA and 50 μM $CdCl_2$ co-treatment.

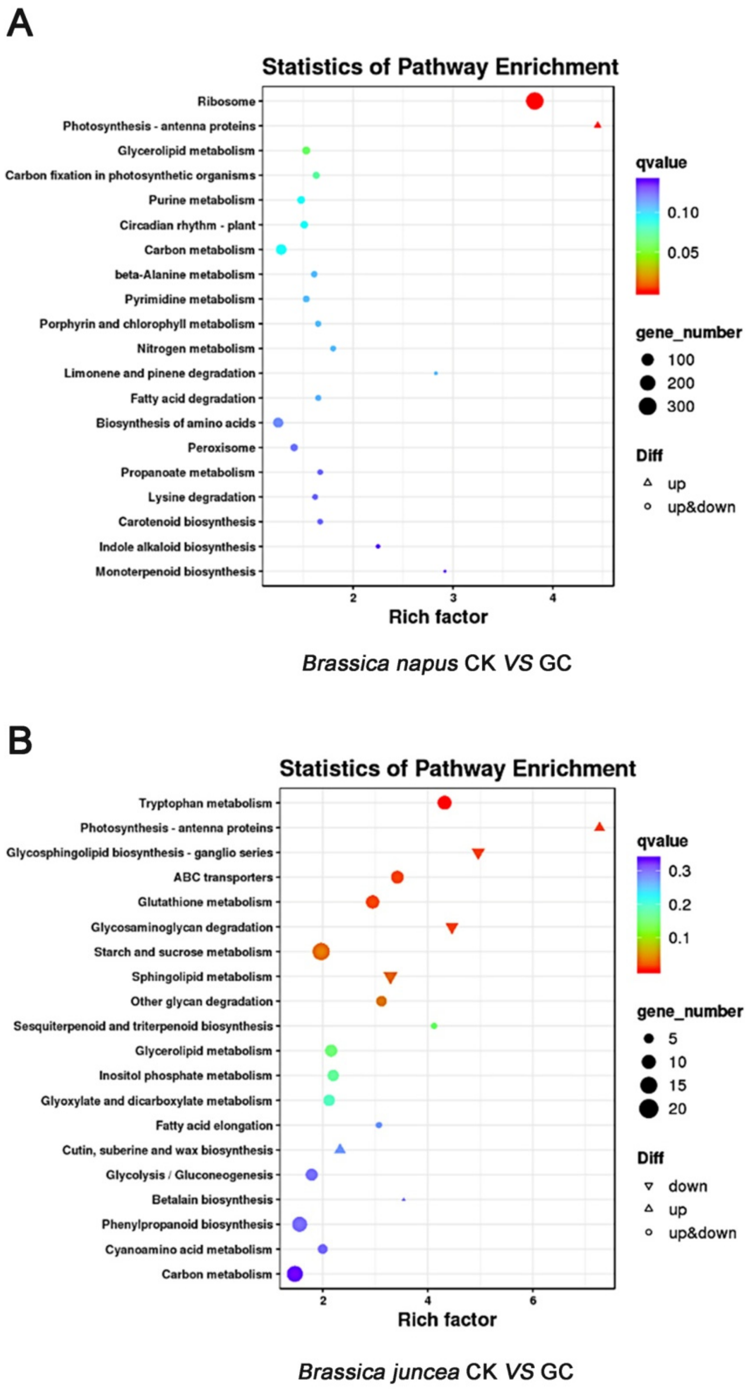

Supplementary Figure S2. KEGG pathway enrichment analysis of differentially expressed genes (DEGs) in *Brassica napus* and *Brassica juncea* under Cd stress and exogenous GABA treatment.(A) KEGG pathway enrichment under Cd treatment alone (CK vs Cd); (B) KEGG pathway enrichment under combined GABA and Cd treatment (CK vs GC). The x-axis represents the rich factor, reflecting the ratio of DEGs to all annotated genes within the pathway. The y-axis lists the KEGG pathway names. The color of the dots indicates the q-value, with smaller values (closer to blue) representing more significant enrichment. The size of the dots denotes the number of DEGs enriched in the corresponding pathway. The shape of the dots represents the direction of gene expression regulation (Diff: upward triangle for up-regulated, downward triangle for down-regulated, and circle for both up- and down-regulated).

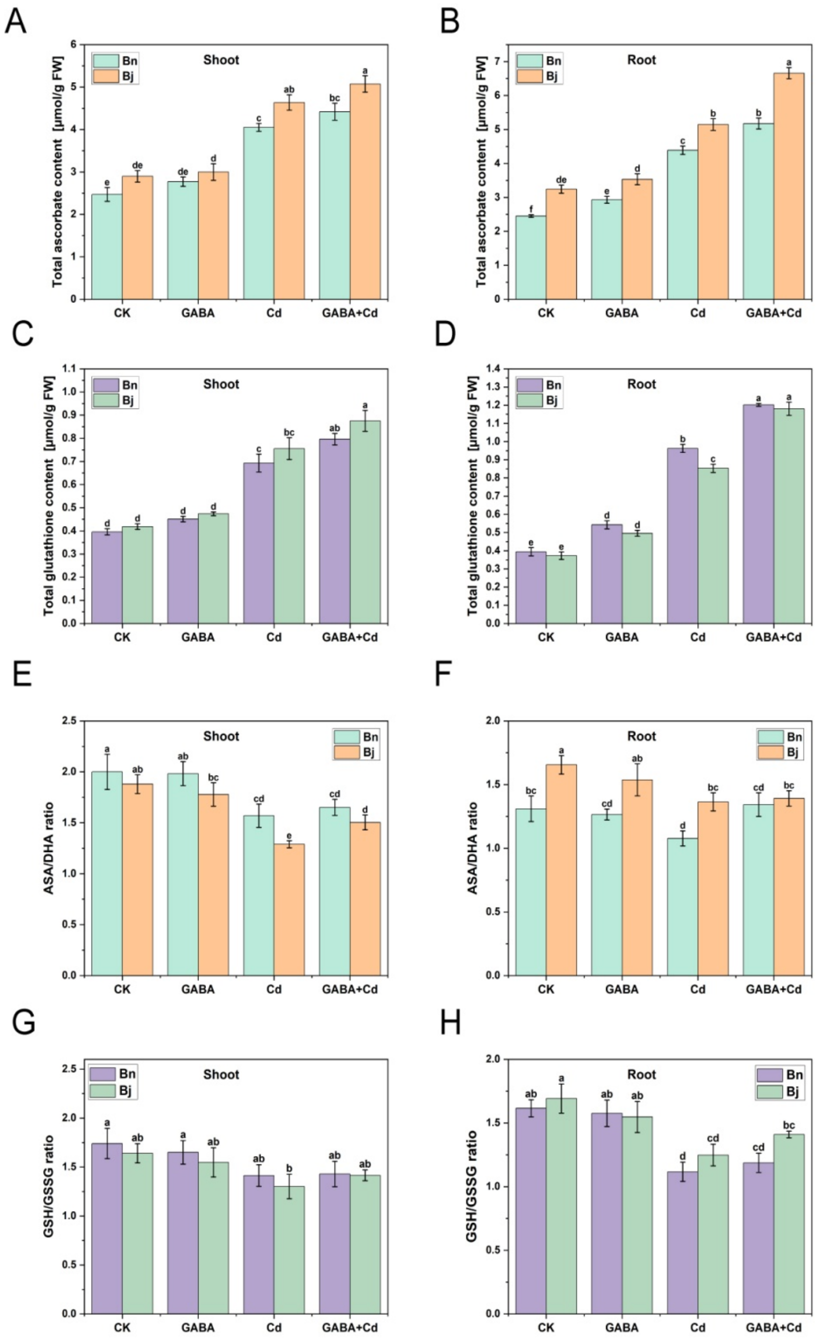

Supplementary Figure S3. Effects of GABA treatment on GSH and ASA redox status of *Brassica* species seedlings under Cd stress. Total ASA contents in shoots (A) and roots (B). Total GSH contents in shoots (C) and roots (D). Ascorbate (ASA)/dehydroascorbate (DHA) ratios in shoots (E) and roots (F). Glutathione (GSH)/oxidized glutathione (GSSG) ratios in shoots (G) and roots (H). *Bn, Brassica napus*; *Bj, Brassica juncea*; CK, control; GABA, 5 mM GABA; Cd, 50 μM $CdCl_2$; GABA + Cd, 5 mM GABA and 50 μM $CdCl_2$ co-treatment. Bars represent means ± SD (*n* = 3). Different lowercase letters indicate significant differences among all treatment × species combinations according to one-way ANOVA followed by Duncan's multiple range test ($P \leq 0.05$).

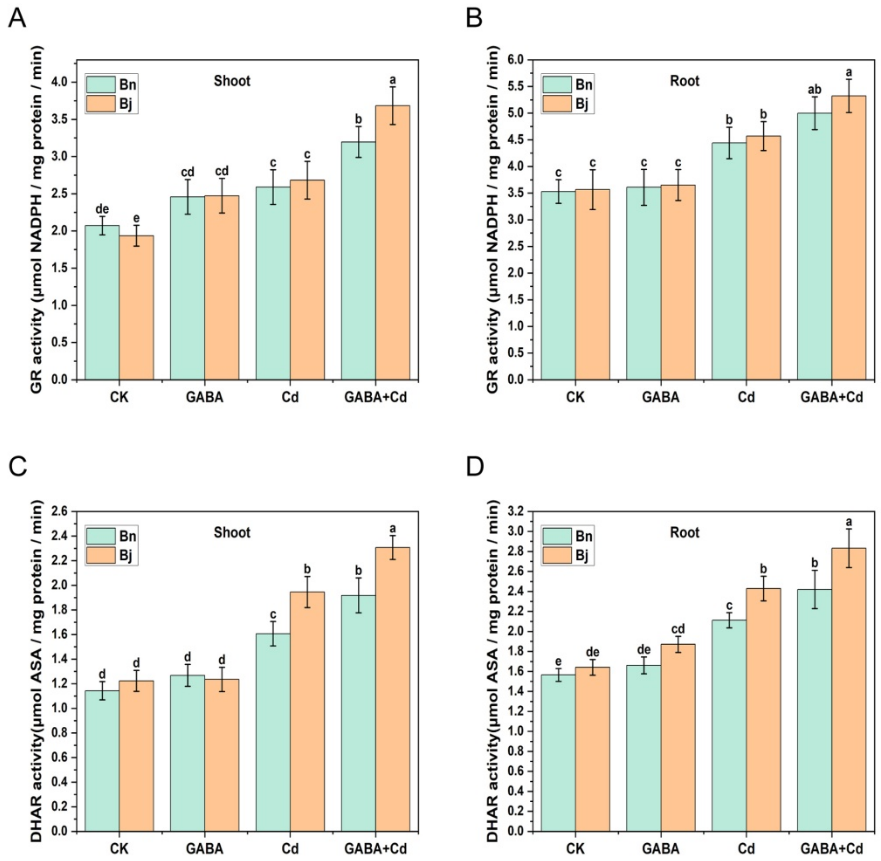

Supplementary Figure S4. Effects of exogenous GABA on antioxidant enzyme activities related to AsA and GSH metabolism in *Brassica* species seedlings under Cd stress. Dehydroascorbate reductase (DHAR) activities in shoots (A) and roots (B). Glutathione reductase (GR) activities in shoots (C) and roots (D). *Bn, Brassica napus*; *Bj, Brassica juncea*; CK, control; GABA, 5 mM GABA; Cd, 50 μM $CdCl_2$; GABA + Cd, 5 mM GABA and 50 μM $CdCl_2$ co-treatment. Bars represent means ± SD (*n* = 3). Different lowercase letters indicate significant differences among all treatment × species combinations according to one-way ANOVA followed by Duncan's multiple range test ($P \leq 0.05$).

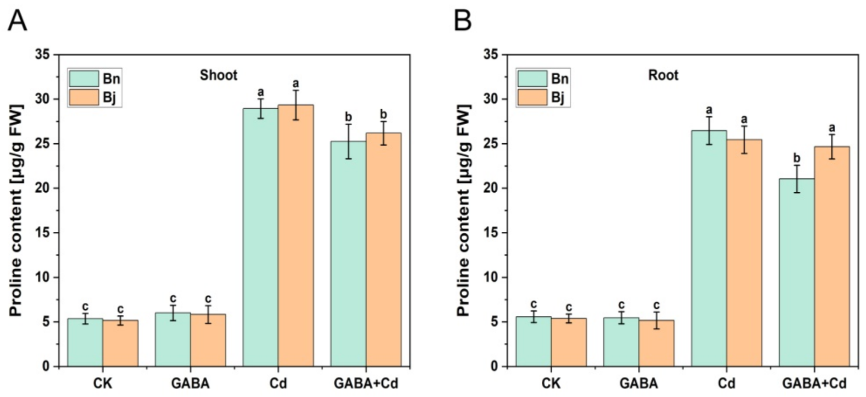

Supplementary Figure S5. Effects of GABA on the content of proline in *Brassica* species seedlings under Cd stress. proline content in shoots (A) and roots (B). *Bn*, *Brassica napus*; *Bj*, *Brassica juncea*; CK, control; GABA, 5 mM GABA; Cd, 50 μM $CdCl_2$; GABA + Cd, 5 mM GABA and 50 μM $CdCl_2$ co-treatment. FW: fresh weight. Bars represent means ± SD (*n* = 3). Different lowercase letters indicate significant differences among all treatment × species combinations according to one-way ANOVA followed by Duncan's multiple range test ($P \leq 0.05$).

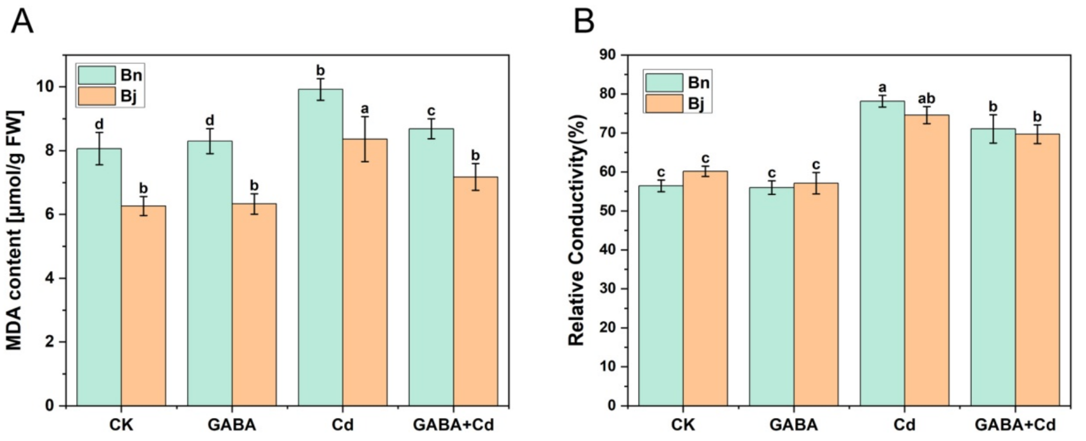

Supplementary Figure S6. Effects of GABA on membrane lipid peroxidation damage in *Brassica* species seedlings under Cd stress. (A)MDA content. (B) Relative electrical conductivity. *Bn, Brassica napus; Bj, Brassica juncea*; CK, control; GABA, 5 mM GABA; Cd, 50 μM $CdCl_2$; GABA + Cd, 5 mM GABA and 50 μM $CdCl_2$ co-treatment. Bars represent means ± SD (*n* = 3). Different lowercase letters indicate significant differences among all treatment × species combinations according to one-way ANOVA followed by Duncan's multiple range test ($P \leq 0.05$).

Table S2. Subcellular distribution of Cd in shoots and roots of different varieties under Cd stress.

Varieties	Treatments	Cell wall (mg/g DW, %)	Organelle (mg/g DW, %)	Soluble fraction (mg/g DW, %)
Bn shoot	Cd	9.15±1.47b (46.29%)	1.85±0.3a (9.34%)	8.77±1.41ab (44.37%)
	GABA+Cd	6.76±1.3b (43.80%)	1.01±0.07b (6.55%)	7.66±1.48b (49.65%)
Bj shoot	Cd	12.58±2.11a (49.33%)	1.16±0.2b (4.55%)	11.76±1.97a (46.12%)
	GABA+Cd	9.2±1.08b (45.61%)	0.51±0.06c (2.52%)	10.46±1.23ab (51.87%)
Bn root	Cd	48.15±5.1b (41.03%)	11.41±1.21a (9.72%)	57.8±6.12b (49.25%)
	GABA+Cd	31.83±2.31c (38.59%)	7.11±1.06b (8.62%)	43.56±2.72c (52.8%)
Bj root	Cd	70.62±9.01a (46.40%)	5.51±1.7b (3.62%)	75.98±9.69a (49.95%)
	GABA+Cd	39.03±2.48bc (42.51%)	2.78±0.83c (3.03%)	50.04±3.18bc (54.48%)

Table S3. The PCR primers sequences.

Gene	Location	Forward primer	Reverse primer
DHAR1	BnaA06g13760D	TAATGGACCACTGGTTTGGTTT	CTAATGGCTAAGAACCACTGAGG
GPX6	BnaA02g21680D	GTGGGGTTTTTGGAGACGGA	TTCTTCAGGTCCTTCTCAATGC
GPX3	BnaC03g24130D	AGGCGGATTGTTTGGGGATT	CTTCTCCTCTCCTGTCCTTGAA
VTC2	BnaA03g48310D	CAGACTGCTGTGTTTGCCTTC	TCTCTGCGTAACACTGTGGC
GSTF2	BnaC03g30880D	TCTTTCCTATCCCACAACCCTT	CTTGGCCTTCGTATCGGTGA
GSTU13	BnaA09g29540D	CCAACGAGAAACACGTCTCC	GCATAAGGGCTCGACCAAGA
ABCG21	BnaCnng21050D	AACGGTCAATGGATCGAGAG	ACAGTAGACCCACCTGATCTT
ABCG15	BnaC05g31240D	GTTGCTCCAAAGGCTAAACGG	GTTTCTTGCGAGTCTACCTGC
ACTIN	LOC106390277	AATGGTGAAGGCTGGTTTTGC	GTATTTCCTCTCAGGCGGTGC
